# Insights on the Generation of Polymeric Drug Carriers via Supercritical Fluid-Assisted Technologies

**DOI:** 10.3390/ma19183825

**Published:** 2026-09-08

**Authors:** Alessandra Zanotti, Stefano Cardea

**Affiliations:** Department of Industrial Engineering, University of Salerno, Via Giovanni Paolo II, 132, 84084 Fisciano, Italy; azanotti@unisa.it

**Keywords:** polymeric drug carriers, drug delivery, supercritical fluids, aerogels, porous structures, microparticles, nanoparticles, capsules

## Abstract

Polymeric drug carriers have emerged as versatile platforms for improving therapeutic efficacy, drug stability and controlled and sustained release of active pharmaceutical ingredients. Conventional manufacturing techniques often rely on high processing temperatures, utilization of organic solvents and consequent purification procedures that may compromise morphology, encapsulation efficiency and carrier biocompatibility. Supercritical fluid technologies overcome the limitations of conventional methods and emerge as environmentally friendly, sustainable and flexible alternatives to produce advanced polymeric drug carriers. This review provides insights into the most widespread supercritical fluid technologies, encompassing the most recent advancements (2022–2026) made in the field of porous materials, micro- and nanoparticles and microcapsules. Emphasis was placed on carrier characteristics, features and applicative translation; this review aims at evidencing advantages, current limitations and future perspectives of supercritical fluid technologies, aiming at encouraging technological and industrial advancement of next-generation advanced polymeric drug carriers.

## 1. Introduction

Drug Delivery Systems (DDSs), defined as formulations or devices that allow controlled and/or targeted introduction of Active Pharmaceutical Ingredients (APIs) into the human body [1,2], are being rapidly affirmed as fundamental pillars of biomedical and pharmaceutical sciences: indeed, DDSs are meant to improve drug stability, bioavailability, therapeutic precision, and patient compliance while avoiding adverse side effects altogether [3,4,5].

Numerous kinds of conventional (tablets, capsules, powders, oral suspensions, ointments and creams, etc. [6,7,8,9,10]) and innovative (micelles, lipidic and inorganic nanoparticles, dendrimers, carbon-based nanomaterials, etc. [11,12,13,14,15]) DDSs have been proposed in the scientific literature. Even though conventional platforms are well-established on the market, they are not devoid of issues, namely poor selectivity and efficacy at low dosages, necessity to administer or apply the drug twice or more during the day, burst releases and difficult control over the therapeutic window [16,17,18,19]. On the other hand, innovative strategies are designed as improved tools for drug delivery, but most of them raise questions of toxicity, apart from being unstable and sensitive to light and oxygen [20,21,22,23,24].

As alternative solution to overcome such limitations, Polymeric Drug Carriers (PDCs) sit at the forefront of the most recent advancements in the field. PDCs are characterized by a polymeric backbone that accommodates, protects and eventually delivers an API [25,26,27]. Undoubtedly, the key to PDCs’ success is their versatility when compared to conventional delivery platforms: simply leveraging polymer concentration, composition and processing workflow, it is possible to swiftly transition from 3-D porous materials to micro-/nanoparticles, allowing fine-tuned stimuli-responsiveness, degradation and/or erosion profiles, release kinetics, cellular and tissue selectivity, etc. [28,29,30,31,32]. Furthermore, the field of pharmaceutical and biomedical sciences also calls for carrier biocompatibility and low toxicity. In this regard, biopolymers—either of natural or synthetic origin—comply with such strict requirements. Both natural (e.g., chitosan, alginate, cellulose and its derivatives, carrageenan, starch, etc.) and synthetic (e.g., poly(ethylene glycol)—PEG, poly(lactic acid)—PLA, poly(lactic-co-glycolic acid)—PLGA, poly(ε-caprolactone)—PCL, etc.) polymers can serve as viable platforms for advanced therapeutic strategies [33,34,35,36,37,38,39].

**Figure 1:**
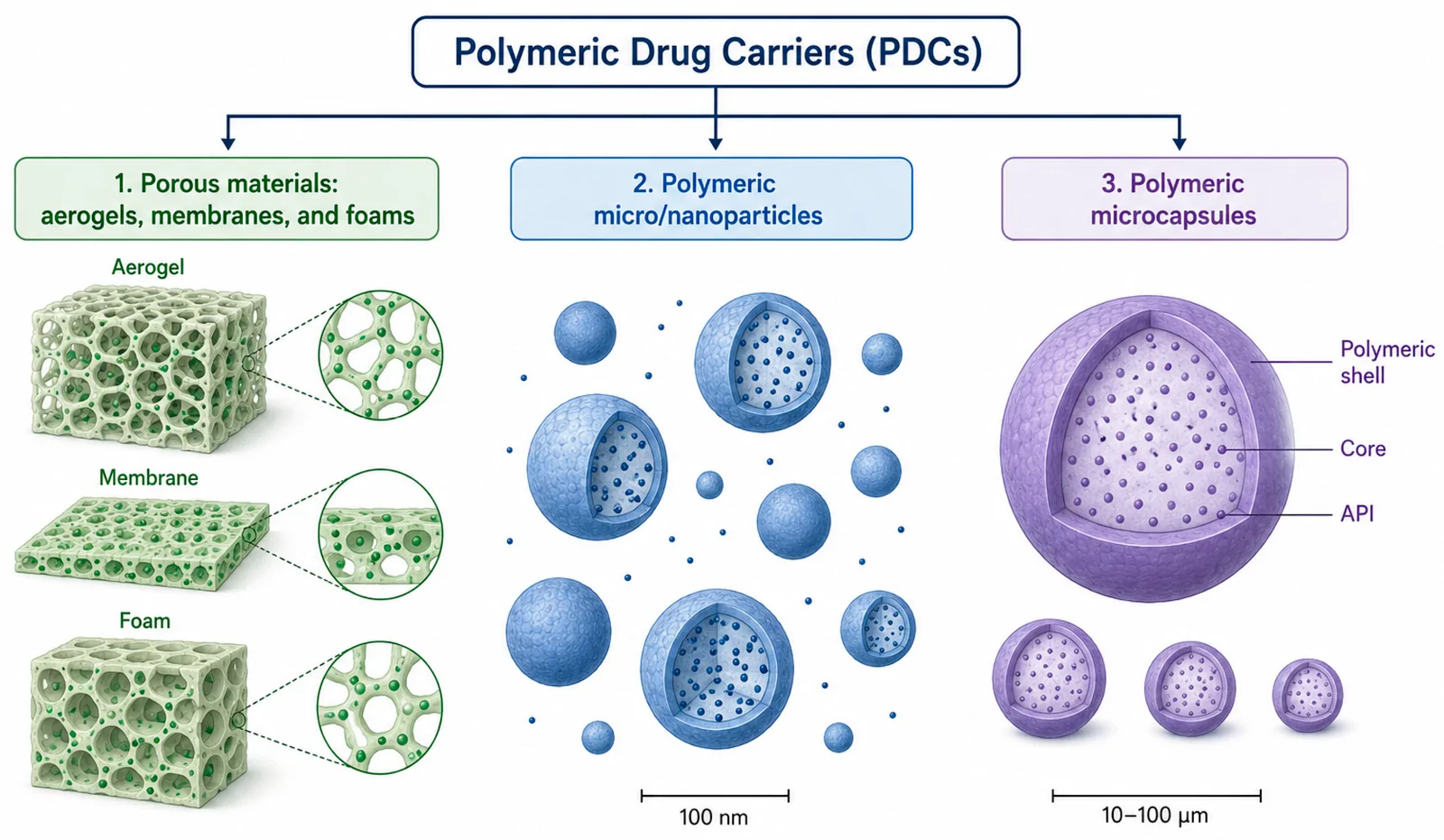
Representation of main PDCs.

In what follows, focus will be placed on the following kinds of PDCs (the basic distinctions between these vehicles are qualitatively depicted in Figure 1, although a more in-depth description will be provided later on):

1. Porous materials: aerogels, membranes, and foams;
2. Polymeric micro-/nanoparticles;
3. Polymeric microcapsules.

Despite the remarkable advancements recently made on PDCs, their practical translation is still challenging due to a combination of manufacturing, regulatory, and economical issues. Specifically, conventional processes often involve the utilization of organic solvents, imposing the subsequent removal from the formulation: apart from obliging the manufacturer to design post-processing steps, this procedure results in drastic morphological variations, loss of API effectiveness upon heating and possible solvent residue in the final product [40,41,42,43]. Clearly, these drawbacks result in the incomplete development of PDCs, limiting the exploration of their full potential. In this frame, supercritical fluid technologies come into play as an auxiliary, but essential toolbox, to generate advanced biopolymeric drug carriers that overcome the bottlenecks of the conventional processes. Supercritical fluids uniquely behave in between gases and liquids: they feature gas-like diffusivities and surface tension, and liquid-like density and solvent-power [44,45]. Among the eligible supercritical fluids, carbon dioxide is the by far most practical one: it has mild critical conditions (31.1 °C and 73.8 bar, which avoids degradation of thermosensitive compounds), and it is easily accessible, low-cost, and non-toxic [46,47]. However, the most interesting feature of supercritical carbon dioxide (SC-CO_2_) is by far its tunable behavior: it can act as drying medium, solvent, cosolvent, or antisolvent depending on the operating conditions and the process layout [48,49,50]. Although supercritical fluid technologies are theoretically superior to conventional processes, their exploration is still mostly confined to the laboratory scale: the high cost of high-pressure plants is still the major concern that undermines the development of these innovative processes on a large industrial scale.

In light of these considerations, this review aims at highlighting the strengths of supercritical carbon dioxide-based techniques in the high-end pharma/biomedical field, paving the way for a newer, more sustainable processing strategy. Although there are several reviews on the topic of supercritical fluid application in the pharmaceutical and biomedical fields [51,52,53], the present work aims at contributing to the base of knowledge by analyzing the most recent literature on the topic, while providing a comprehensive summary of the main principles underlying biopolymeric drug carrier generation and their effect on final performance. This review will be organized as follows: the PDCs of interest—mainly biopolymeric—will be described, and the conventional technologies proposed as well; for each product, supercritical fluid-based alternatives will be discussed in depth, evidencing strengths and weaknesses; eventually, applicative performances will be evaluated, and the formulation placed in a well-defined practical scenario to facilitate the introduction of novel materials in large-scale applications.

## 2. Materials and Methods

The bibliographic study focuses on the most recent scientific literature: works published in the period 2022–2026 were considered to address the established progress made in this field. ChatGPT was employed for the sole purpose of generating or correcting some artwork.

## 3. Porous Materials

Aerogels, membranes and foams fall within the variegated category of porous materials.

### 3.1. Aerogels

Aerogels are an advanced class of 3-D nanostructured materials, characterized by the existence of a complex and interconnected porous architecture that ranges from the macroscale to the nanoscale [54]. This unique spatial configuration allows aerogels to feature high porosity values (>90%), large specific surface area (SSA, 10^2^–10^3^ m^2^/g), and ultralow density (<0.1 g/cm^3^) and thermal conductivity (down to 0.01 W/m·K) [55,56,57]. Among the porous structures, aerogels are the ones that encountered the most success in the pharma/biomedical field: in this context, they are used mainly as drug reservoirs due to their high pore volume. Indeed, it is possible to achieve high drug loadings in small volumes, but also to control or sustain drug release rates thanks to the tortuous porous network to be overcome during diffusion towards the external aqueous bulk [58].

The aerogel production workflow begins with the sol–gel route, during which a biopolymeric colloidal suspension responds to an external trigger (pH, temperature variations, addition of crosslinkers or catalysts, etc.), rearranging in a unique three-dimensional configuration, depending on the structuring material and the gelation mechanism. The result of this rearrangement is the so-called “gel”, which is a biphasic system made up of a solid skeleton rich in the fluid of the native sol [59]. When preparing biopolymeric gels, the fluid is generally water; thus, the gels are referred to as “hydrogels”. However, aerogels are dry materials, hence the necessity to remove water from the hydrogel. Conventionally, this step can be achieved thermally, either by evaporation (air drying or oven drying) or sublimation (freeze drying). Even though these procedures are somehow simple in nature, they result in the complete loss of the hydrogel nanostructure. During air drying or oven drying, the capillary stress exerted on the nanopore walls by the liquid–vapor meniscus leads to structural collapse; hence, a nonporous material is obtained [60]. Moreover, if the gel is loaded with an active compound, the heat load or oxidative stress defies the purpose of generating a stable and functional drug carrier. On the other hand, freeze drying involves a sublimation step, namely, the hydrogel is subjected to extremely low temperature (−50 °C) and high vacuum. This combination favors the formation of ice crystals (whose dimensions are dictated by the crystallization rate) that act as templates to the final porous structure. This process results in the formation of highly porous materials, but devoid of the nanostructure; therefore, low specific surface areas are obtained, and the release rate cannot be easily tuned [61,62].

**Figure 2:**
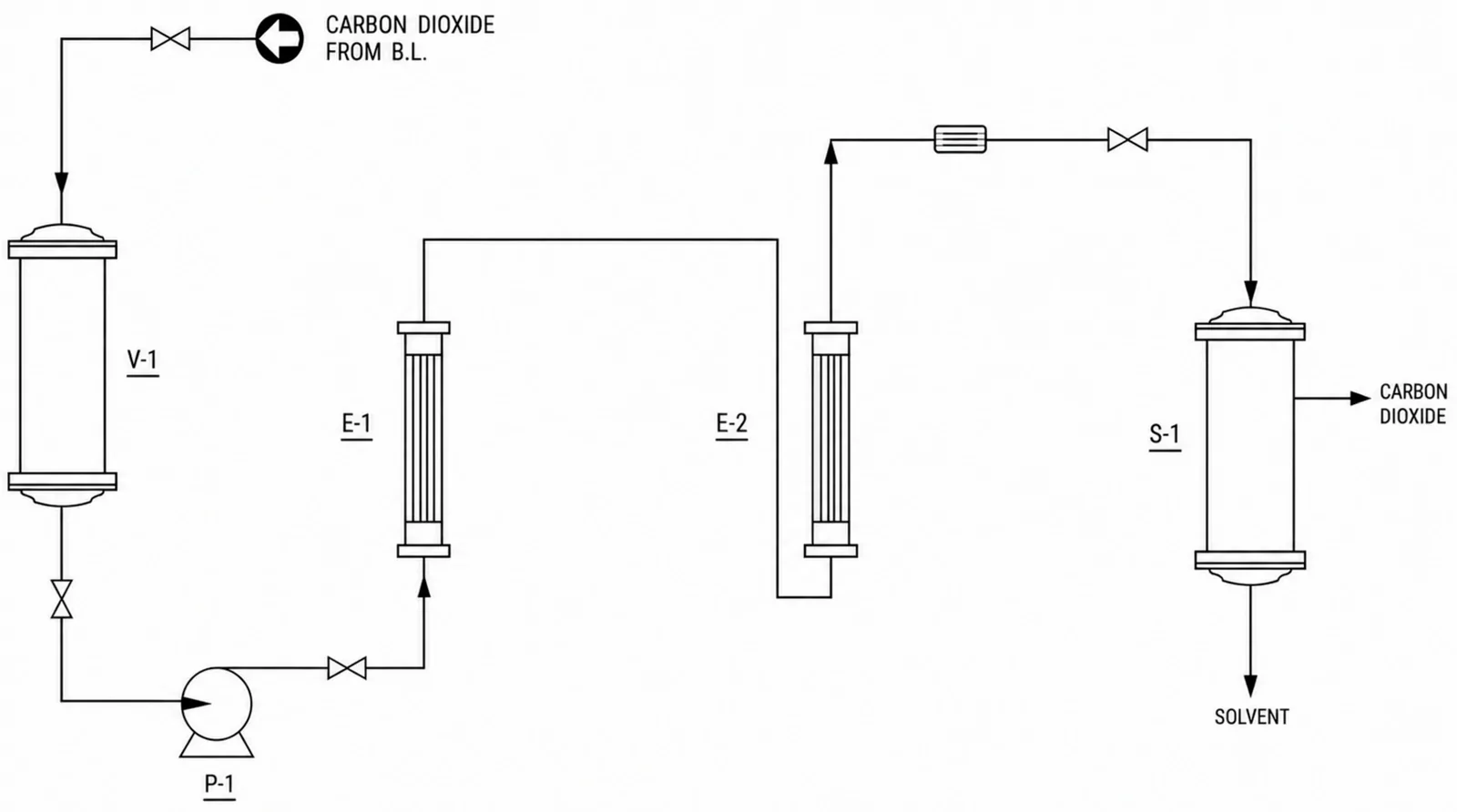
SGD plant scheme and its main elements: V-1, refrigerating bath; P-1, high-pressure pump; E-1, mixer; E-2, high-pressure vessel; S-1, separator.

In contrast with these established drying methods, supercritical gel drying (SGD) consists of an extraction mediated by supercritical CO_2_, provided that water is substituted with a fluid miscible in CO_2_ (i.e., a solvogel must be obtained). In such circumstances, SC-CO_2_ acts as drying medium: it fills the solvogels’ pores, creates a supercritical mixture with the solvent embedded in the gel, and counterdiffuses from the porous structure. Eventually, the dry aerogel is obtained: it virtually preserves the native gel morphological architecture due to supercritical carbon dioxide near-zero surface tension, which allows for a rapid and efficient extraction [63,64]. For the sake of example, a representative scheme of the SGD plant of our research group at the University of Salerno is reported in Figure 2.

With a view to a possible scale-up, it should be considered that SGD is intrinsically a batch process (i.e., a solid–liquid extraction): to improve productivity, multiple extractors could be lined up to work in parallel, so that while a vessel undergoes depressurization, the others can alternatively operate. Even though the costs of working at high pressures are non-negligible, it should be considered that the differences between aerogels and other kinds of gel-derived porous structures are remarkable: in this case, the cons of operating high-pressure equipment are outbalanced by the benefits that arise from the quality of the final product. In addition, aerogels’ shape can be designed depending on the final purpose: they can be monoliths, thin membranes, beads or microparticles [65,66]. For instance, monolithic or thin membranes can be intended for wound dressing and wound healing, or might be even used as advanced tablets [67,68,69]; beads are eligible for oral drug delivery [70,71,72], while microparticles can be used for both oral drug delivery and as inhalable powders [73,74]. So, aerogels constitute flexible porous platform whose design can be engineered for a specific purpose.

It should be also considered that aerogels can—and must—be loaded with an active compound: drug loading can be carried out during different steps of the production procedure, namely [58]:

1. During the gelation step: Hydrophilic compounds can be mixed with the precursor gel and then undergo gelation altogether.
2. During the solvent exchange procedure: In the case of species poorly soluble in pure water (e.g., polyphenols), they can be solubilized in the exchange solvent, and thus impregnated. However, during SGD, SC-CO_2_ and the solvent embedded in the solvogel form a supercritical mixture that might enhance active compound solubility and its extraction from the porous structure. In this case, effective drug loading efficiency might not be considerably high, depending on API solubility in the mixture.
3. After drying, recurring to supercritical fluid impregnation (SFI) when the compound is fairly soluble in carbon dioxide. This alternative is actually more performative than the second scenario, since it leverages SC-CO_2_ oversaturation with an active compound, which is thus diffused and homogeneously precipitated inside the porous structure upon depressurization.
4. Performing wet impregnation after drying: In this scenario, it is safe to assume that most of the drug can diffuse inside the porous structure, but at the expense of the morphological architecture. Indeed, after wet impregnation, the liquid must be removed by evaporation, leading to partial nanostructure collapse due to capillary stresses.

**Figure 3:**
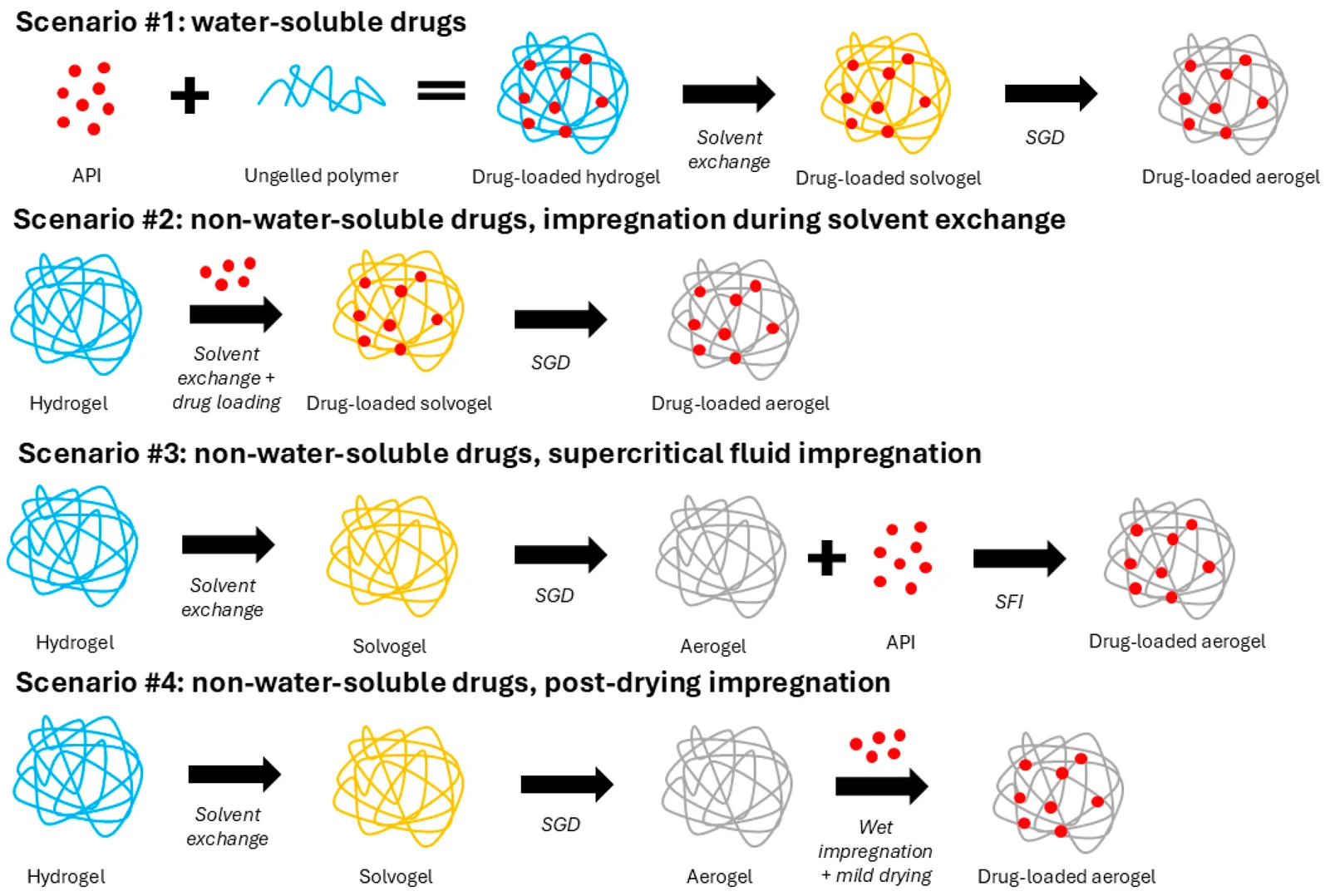
Different drug loading procedures on aerogels.

A representative scheme is reported in Figure 3.

There are numerous, yet variegated examples of biopolymeric drug carriers reported in scientific literature. For instance, Menshutina et al. [75] proposed clomipramine-loaded chitosan aerogel microparticles as nasal drug delivery systems: while comparing two different formation mechanisms, i.e., emulsion gelation and spray drying, they noticed that aerogels’ textural properties (pore volume, SSA, bulk density, etc.) were not significantly affected by the atomization procedure. However, the aerodynamic diameter was considerably lower for aerogels prepared via emulsion gelation rather than spray drying, suggesting that emulsion-derived particles are less prone to aggregation. It is worth noting that aerogel particles with a larger mean average diameter can be intended for nasal drug delivery, while the smaller ones can be considered for inhalation: indeed, a lower aerodynamic diameter allows particles to be conveyed more easily to the rest of the respiratory trait. The authors achieved a mass loading of 35%*w*/*w*, employing a post-drying wet impregnation procedure. Even though there are no tests in vitro, drug administration tests on rats showed that clomipramine reached the brain and the blood rapid and selectively, showing good therapeutic potential. Clomipramine concentration was at a maximum in plasma after 10 min, and in the hippocampus after 30 min. Similarly, clomipramine was impregnated, using the same procedure as before, on aerogel microparticles obtained from egg white proteins [76], achieving a maximum loading efficiency of 14.88%*w*/*w* (lower than chitosan aerogel microparticles), indicating that gel chemistry, porosity and density affect loading efficiency. Indeed, chitosan microparticles feature porosity values up to 99.2%, while protein-based ones reach 85%, even though pore volumes and SSA values were comparable (1.49 vs. 1.6 cm^3^/g, and 254 vs. 275 m^2^/g). In this case, it was concluded that clomipramine concentration spiked in the hippocampus after 10 min, spiking after 30 min in rat plasma. The values are inverted for chitosan and egg white-based aerogels, indicating that differences in drug release can be attributed to the nature of the biopolymeric carrier. Alnaief et al. [37] synthesized hybrid alginate-based polysaccharidic aerogel microparticles via emulsion gelation and supercritical drying, and loaded ibuprofen using SFI. The combination of these two supercritical techniques allows for high drug loading efficiency (up to 93.5%), and an effective ibuprofen loading of up to 18.7 ± 1.1%. Interestingly, in this case the goal was to anticipate drug release, in such a way that ibuprofen—which is notably an anti-inflammatory drug—could be accessed more easily: indeed, the choice of the nature of the carrier (i.e., alginate, pectin, carrageenan, or mixtures) enables this task since biocompatibility is enhanced, although its shape (i.e., microparticles) also induces rapid drug solubilization kinetics. Méndez et al. [77] used different citrus pectin to generate aerogel microparticles, eventually loaded with vanillin using SFI. They eventually concluded that vanillin release is affected by aerogel textural properties, but also by the presence of hydrophobic groups; namely, the more the hydrophobic groups in the aerogels, the slower the release of the active compounds. Moreover, if aerogels feature smaller pore size, solvent intrusion is hindered, hence delaying drug release. Nevertheless, they proved that it is possible to vehicle hydrophobic compounds by combining two supercritical fluid-based techniques. Suttiruengwong et al. [78] obtained pectin aerogel beads, subsequently loaded with ibuprofen via SFI. However, the authors point out that ibuprofen is only slightly soluble in SC-CO_2_; hence, a co-solvent is needed (i.e., ethanol). On this aspect, there is an apparent minor disagreement with Alnaief et al. [37], who did not report the utilization of co-solvents to enhance ibuprofen solubility, but it is also to be considered that there are different mass transport phenomena involved in microparticles or beads (i.e., resistances). Contrary to the aforementioned work, in this case ibuprofen release was delayed with respect to pure ibuprofen powder up to 20 times. The choice of the structuring biopolymer, but also the carrier dimensions, influence release profile. Indeed, aerogels can be engineered in terms of shape depending on the desired outcome (i.e., anticipated or delayed drug release). Sellitto et al. [79] generated silk fibroin–alginate hybrid aerogel beads at different relative polymer ratios. Ciprofloxacin was loaded in the precursor sol prior to the gelation step. The aim of the work was to obtain hemostatic dressings: aerogel fluid uptake capacity was up to about 400%, but a significant weight loss was appreciated after 40 min. Nevertheless, the hybrid aerogels broke down after 56 days, while half of the mass was lost after one day: such findings suggest that aerogels, if properly synthesized, are stable and comfortable structures eligible for long-term applications. Ciprofloxacin in vitro release tests show that the drug is conveyed gradually over 24 h, while outperforming commercial gauzes in terms of coagulation potential. Illanes-Bordomás et al. [80] advanced the idea of aerogel beads by creating core–shell formulations based on alginate and konjac glucomannan. They loaded either vancomycin or dexamethasone base, soluble and non-soluble in water, respectively, prior to the gelation step; hence, a solution or a dispersion was obtained. Interestingly, dexamethasone release from the aerogels was anticipated with respect to pure powder solubilization, whereas the trend was inverted for vancomycin. This finding evidences the dual nature of aerogels as drug carriers: the same formulation allows better dispersion and amorphization of poorly soluble drugs, thus facilitating more effective release; on the other hand, if the API is fairly soluble in water, the limiting step lies in overcoming porous network tortuosity. Nevertheless, in both cases the majority of the drug is released in short times (i.e., about 1 h). Behruzi et al. [81] produced monolithic starch aerogels, while ibuprofen loading was achieved after the drying stage using a suitable mixture of isopropanol and ethanol. The result of this procedure was an anticipated drug release in PBS at pH 7.4, while slightly anticipated at pH 1.2. Being that the characteristic length of the carrier is clearly higher than beads or microparticles, ibuprofen release took up to 400 min, indicating the versatility of aerogels in controlling release rate depending on the desired therapeutic outcome. Even though drug loading was achieved by means of sequential wet impregnation, it is safe to assume that the utilization of organic solvents for this purpose should be limited or monitored if the final product is meant for pharmaceutical applications; in addition, post-supercritical drying evaporation to allow drug precipitation might interfere with the native porous structure. Overall, drug release might even be fine-tuned if the nanostructure is preserved during impregnation. Also, Mottola et al. [82] attempted to synthesize starch aerogels, subsequently loaded with quercetin by SFI. They observed that quercetin release depended on starch concentration, allowing them to reach the plateau value after 140 min using a 30%*w*/*w* starch aerogel. Moreover, these devices exhibited a fungistatic effect, evidencing their potential as ready-to-use materials in pharmaceutical applications, overcoming the bottleneck of releasing poorly soluble drugs. Yu and Budtova [83] explored the potential of carboxymethyl cellulose aerogels, and compared them with corresponding structures obtained by freeze drying. The most striking, yet expected, result is associated with the calculation of the L-ascorbic acid 2-phosphate diffusion coefficients obtained from in vitro releases and validated via compartmental models: diffusivity across aerogels is four times smaller than the one across cryogels, suggesting that the existence of the nanoporous architecture allows for more controlled releases. However, it should be noted that the final choice of the porous structure depends on the final target: if the goal is a rapid release, a macroporous structure should be considered since it allows rapid water intrusion and drug counterdiffusion; a nanoporous structure is the most eligible choice when it comes to achieving controlled release rates. Al-barudi at al. [84] developed tragacanth/alginate hybrid aerogels: they proved that it is possible to tailor aerogels’ physico-chemical properties by changing the relative biopolymer ratio. Specifically, alginate addition stiffens the biopolymeric network, leading to higher bulk densities, lower porosities, and lower swelling ratio, but enhanced stability. As a general rule, highly hydrophilic polymers like tragacanth tend to swell more, but to lose mass over time. Balancing these aspects, they found that the highest paracetamol loading (i.e., 114.8 ± 1.86 mg/mg_aerogel_) was achieved on the aerogel with a tragacanth-to-alginate ratio of 3:1. In this case, paracetamol was loaded during the solvent exchange step, given its solubility in ethanol rather than water. It is worth noting that release profiles were affected by the alginate addition: paracetamol concentration increased more rapidly when diffusing from tragacanth rather than hybrid aerogels, suggesting that swelling and erosion can lead to an anticipated drug release. In all the proposed cases, paracetamol release is sustained over a 6–7 h timeframe, paving the way for the treatment of chronic pain.

To sum up, aerogels are versatile and effective vehicles for drug delivery: they can be engineered into carriers of antibiotics, anti-inflammatory agents, and bioactive compounds of natural origin. The correct design of the drug carrier, involving the choice of the structuring materials, shape, and affinity with the active compound allows researchers to tackle the issue of controlled drug release: it is possible to anticipate or retard the release rate, providing flexible tools for treating both chronic and acute illnesses. However, this literature analysis evidences the fact that the workflow must be adopted while also considering the possible changes in aerogels’ textural properties: the most interesting features must be preserved during the impregnation step. This is the main reason why the combination of SGD and SFI represents a viable and powerful tool to obtain superior PDCs.

The most relevant aspects emerging from this survey on aerogels are synthesized in Table 1.

Summarizing the observations in Table 1, it emerges that biopolymeric aerogels are a popular choice when it comes to drug delivery. In most cases, the systems are studied for oral drug delivery applications, but their potential surely extends to other kinds of drug administration methods, as proved by several proof-of-concept-evaluating studies. Even though it is not possible to generalize the differences between different aerogels, since the outcomes depend on multiple factors (e.g., loaded drug, impregnation method, structuring polymer, aerogel shape and size, etc.), some general aspects can be underlined:

- Drug release completes according to the following order: monoliths > beads > microparticles, due to different characteristic lengths and diffusion coefficients;
- Drug release can be tailored depending on the combination polymer-drug: it can be anticipated or retarded, depending on the relative affinity between the species;
- Different loading procedures can be adopted, but the utilization of high-pressure technologies for poorly water-soluble species is gaining popularity when compared to other methods due to high drug loading capacities and enhanced solubility.

### 3.2. Membranes

**Figure 4:**
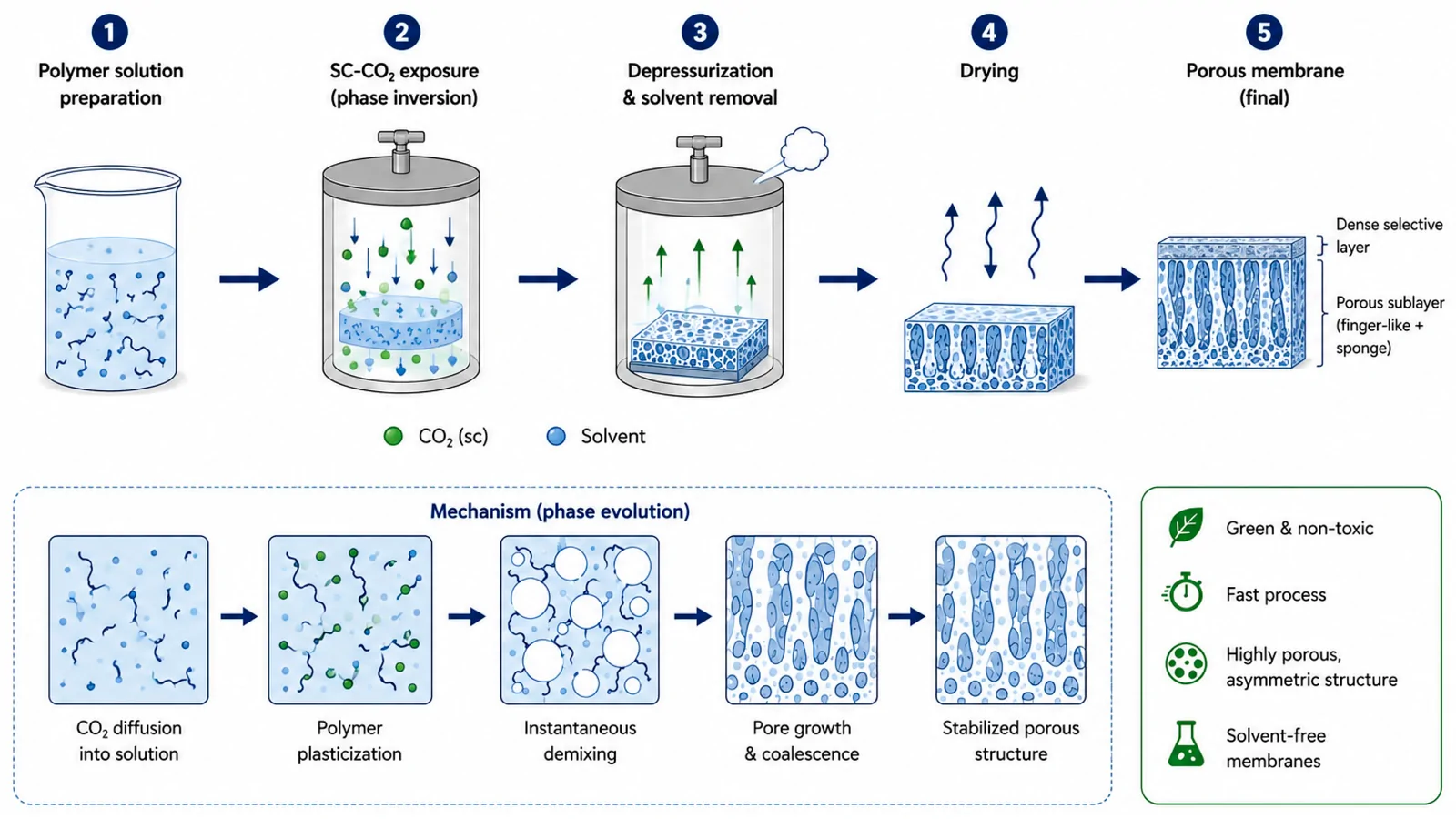
SAPIM mechanism scheme.

Polymeric membranes are macroporous structures that feature oriented, well-defined interconnected pores. Compared to aerogels, membranes are denser and have lower porosity, but they arouse interest in the field of drug delivery due to the possibility of tailoring permeability and selectivity [85]. Moreover, even though a porous structure exists, its degree of three-dimensional interconnection is still lower than aerogels, mainly due to the production method. Indeed, membrane formation depends on thermodynamical instability of a biopolymeric solution when operating conditions change; hence, a phase inversion must be performed. This task can be accomplished thermically (Thermically Induced Phase Separation—TIPS) or using a non-solvent (Non-Solvent-Induced Phase Separation). In the first case, polymer precipitation is induced by increasing temperature and allowing the solvent to evaporate, while in the second case a non-solvent induces thermodynamical instability, and while this liquid–liquid extraction is carried out the polymer precipitates. The NIPS method leverages the relative affinities of the species involved: the polymer is soluble in the solvent and not in the non-solvent, while the original solvent is more affine with the non-solvent than it is with the biopolymer. This dynamic allows the precipitation of the biopolymer, which, depending on the chemical nature of the biopolymers and solvents involved, organizes in a unique porous configuration [86]. Even though these processes are easy to perform, there is poor flexibility in terms of operating conditions, and therefore also in terms of final outcomes. These limitations can be overcome by modifying the NIPS method, employing SC-CO_2_ as non-solvent and a plant layout basically unaltered with respect to SGD. It is worth recalling that supercritical fluid properties can be fine-tuned by changing pressure and temperature, thus density and solvent power: the supercritical fluid-assisted phase separation method (SAPIM) induces the formation of different kinds of morphologies depending on operating conditions. It is possible to move rapidly between the binodal and spinodal curves of the ternary diagram, thus controlling the demixing rate and the final porous morphology [87,88]. A representative scheme of the SAPIM mechanism is reported in Figure 4.

In the past, several works on biopolymeric membranes loaded with different bioactive compounds (e.g., quercetin, curcumin, etc.) showed promising results in terms of controlled drug delivery [89,90,91,92]. For instance, when curcumin is loaded inside cellulose acetate membranes [89], drug release can be sustained for even 70 h, showing controlled and engineered release over time and high antioxidant activity, depending on the phase inversion operating conditions, and hence the porous architecture. Also, cellulose acetate concentration influences the time needed to reach the plateau value. For instance, quercetin release is ten times slower when cellulose acetate concentration increases from 10 to 15%*w*/*w* due to the shift from a finger-like structure to a cellular one [90,91].

The high flexibility and ease of execution should be further exploited in the field, to promote the generation of sustainable ready-to-use PDCs.

### 3.3. Foams

**Figure 5:**
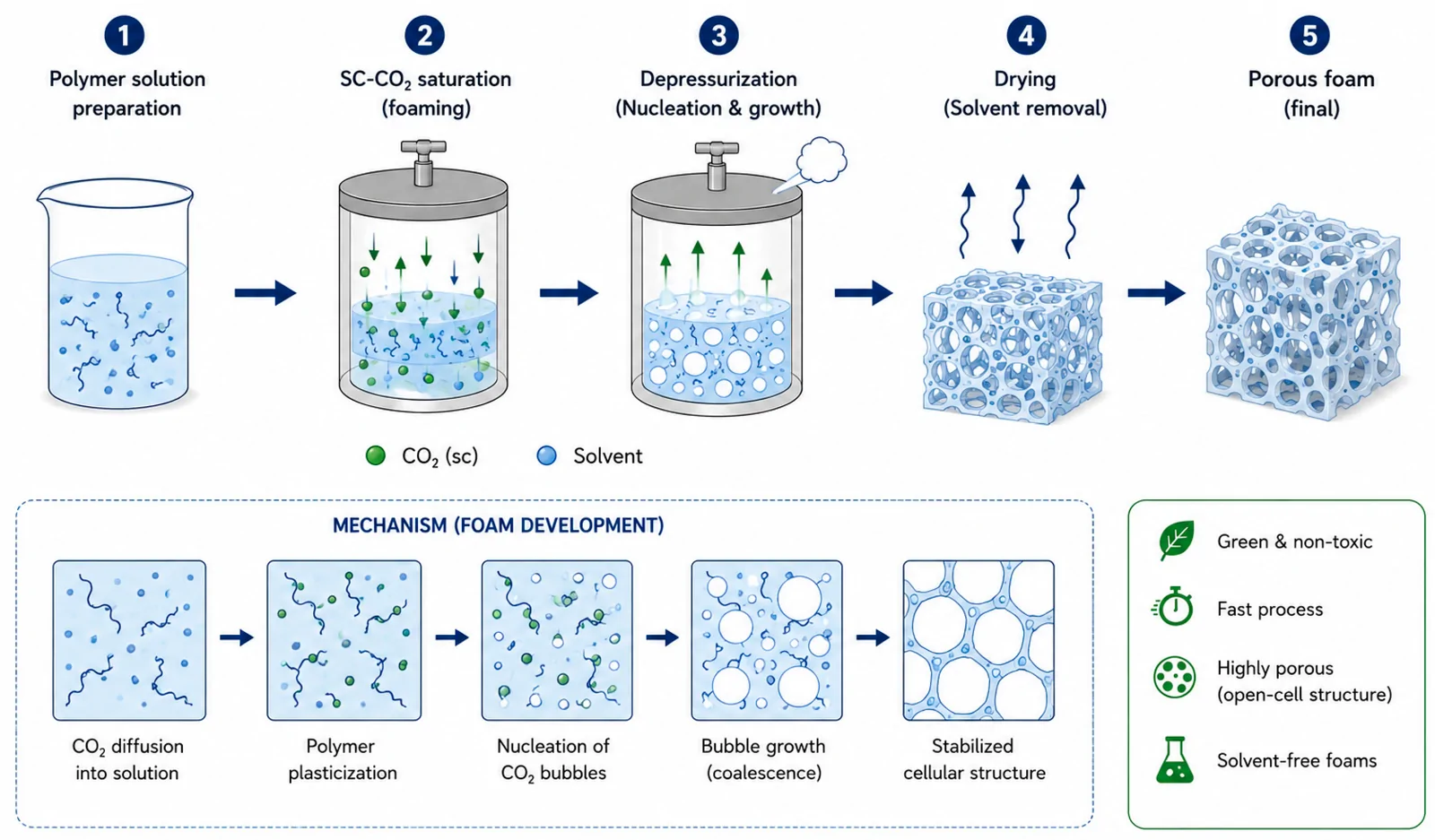
Foam production scheme.

Foams are cellular solids characterized by closed and non-interconnected pores, generated by the nucleation, growth and stabilization of gas bubbles within a molten polymer. Among the conventionally adopted methods to produce foams, chemical or physical foaming is the most widespread [93,94]. Chemical foaming involves the utilization of chemical blowing agents, which basically decompose upon heating and release gases that expand the softened polymer: in this scenario, the most frequently adopted chemical blowing agents are azodicarbonamide, sodium bicarbonate/citric acid systems, hydrazides, carbonates, etc., [95,96], which in most cases generate toxic residues from decomposition and cause poor control over pore size distribution. In addition, the high temperatures involved during heating could damage thermosensitive compounds. Even though this technology is compatible with different polymer processing units (e.g., injection molding, compression molding, extrusion), it might not be always the most eligible choice for biopharmaceutical applications due to potential toxicity of decomposition by-products. Physical foaming, instead, involves the dissolution of a volatile compound inside the polymeric matrix, and its subsequent vaporization [97,98]. Even though it resolves the shortcomings of chemical foaming, many physical blowing agents like alkanes are flammable and might result in the emission of volatile organic compounds (VOCs). In other cases, nitrogen could be used for this purpose. Supercritical foaming is an alternative strategy inspired by physical foaming, and SC-CO_2_ is mostly used for this purpose. It involves the injection of the supercritical fluid in the molten polymer, plasticization, cell nucleation, growth and stabilization upon pressure drop [99,100,101,102]. The process should require up to a few seconds to drop from hundreds of bars to atmospheric pressure to guarantee immobilization of the porous structure upon gas desorption. Even though the fundamentals of supercritical foaming were built on batchwise configurations, this process is also being expanded to continuous setups, confirming the interest that revolves around this approach. The possibility to control pore size distribution, pore gradients and interconnectivity makes supercritical foaming a viable tool for generating PDCs with no solvent residue, allowing the production of fine-tuned drug delivery platforms. A synthetic scheme of foam production via supercritical CO_2_-assisted foaming is reported in Figure 5.

Some examples of supercritical foaming are reported in the scientific literature, most of which are associated with thermal insulation purposes. Nevertheless, some studies focusing on the biopharmaceutical declination of supercritical foams have been recently published. For instance, Satpayeva et al. [103] produced PCL-hydroxyapatite composite foams by combining supercritical foaming and SFI, eventually loading carvacrol with a view to bone tissue engineering applications. Even though they did not perform any in vitro drug release tests, they noticed that it was possible to tailor PCL scaffolds’ porosity and pore size distributions by adjusting the depressurization rate or even by implementing a multiple-decompressing-step configuration. In particular, the double-decompression step allowed them to achieve an impregnation yield of about 10.24%*w*/*w*, suggesting that it is possible to simultaneously foam a polymer and load and API with successful results. Being that the foam porous structure is not as interconnected as that of aerogels, it could be expected that a possible drug release might be hindered by this kind of morphological organization, so it could be an interesting tool for sustained releases over long periods of time. The work of Bendicho-Lavilla et al. [104] confirms this hypothesis. In fact, they produced PLGA intravitreal implants by supercritical foaming, and introduced dexamethasone or bevacizumab as the active compound. Once again, the manipulation of the depressurization rate was the main parameter that drives the definition of morphological organization. However, the most important result is that the drug could be released for up to 4 months, showing incredible promise for the treatment of retinal diseases, leveraging precise release patterns. Moreover, cell viability tests showed that there were no morphological changes in the cells of interest, proving the practical readiness of supercritical foams. Kravanja et al. [105] brought supercritical foaming to coating production, developing a ketoprofen-loaded PLGA bioactive coating on inorganic substrates for orthopedic applications. Ketoprofen release was steady over a period of 5 days, and no dead cells were detected during the cell viability assay. Interestingly, they used tetrahydrofuran as a first deposition step, after which supercritical foaming was performed: even though this is a toxic compound, supercritical fluid technologies are also known for their purification capacity, thus exhibiting multiple beneficial effects. Rivera et al. [106] extruded PLA/PBAT (PBAT—polybutylene adipate terephthalate) blends to obtain polymeric films, subsequently impregnated using SFI and then foamed in a second step. Although there are no release data, the authors evaluated the antioxidant activity, proving that foamed samples exhibit a higher and more sustained activity over time. This behavior is probably due to the correct and homogenous incorporation of caffeic acid inside the porous device, proving also that supercritical foaming is well-aligned with conventional material processing procedures.

Overall, foams are eligible vehicles for prolonged drug release, even for extremely long periods of time. Foaming, in contrast with SGD and SAPIM, can be continuous in nature, making it appealing for a potential scale-up. However, the choice of the porous carrier should depend on the desired target, meaning that it should be defined if drug release kinetics must be instantaneous, prolonged, retarded, or sustained. In addition, this first literature analysis confirms the multifaceted potential of supercritical fluid technologies, which can be declined—to this point, even using the same plant configuration—to obtain devices with different kinds of morphology, depending on the specific application.

## 4. Polymeric Micro-/Nanoparticles

**Figure 6:**
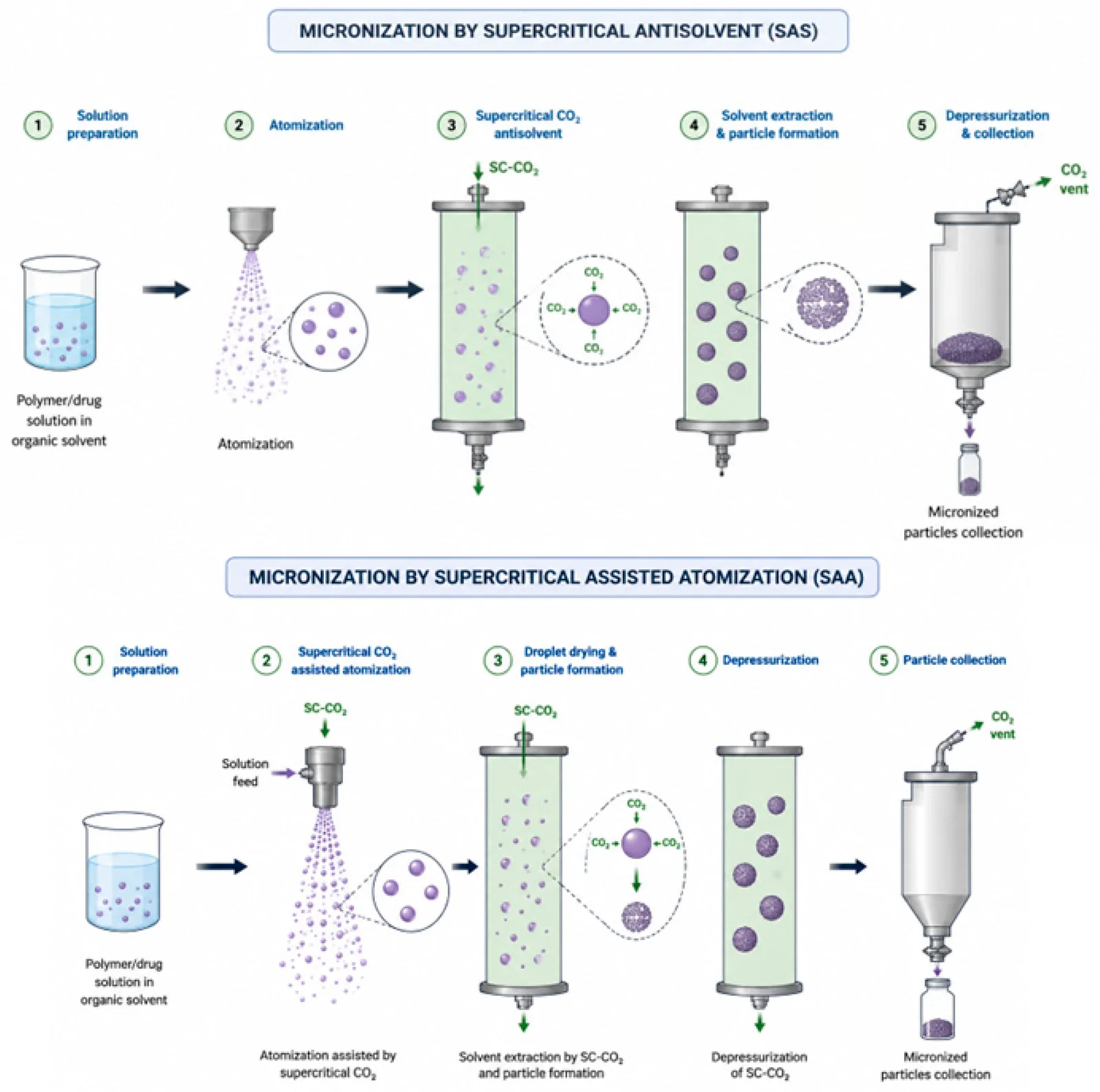
SAS and SAA processes.

Polymeric micro-/nanoparticles are advanced drug delivery systems, composed of biodegradable or biocompatible polymers, and their size ranges from about 100 nm to several hundred micrometers [107]. Recalling the scheme reported in Figure 1, polymeric micro- and nanoparticles are essentially spherical objects made of a continuous polymeric dispersing matrix, and an API homogenously distributed in it. To produce polymer/drug particles, several processes can be employed, like spray drying, jet-milling, solvent evaporation or coacervation [108,109]. Generally, this kind of drug vehicle is produced by preparing a drug + polymer solution and then removing the solvent from it. This separation can be achieved by solvent evaporation or liquid–liquid extraction, but the problem lies in the utilization of high temperatures, which might interfere with thermosensitive compounds, or potentially toxic organic compounds that must be eventually removed. Indeed, large amounts of solvents must be used, and their residues in the final formulation are non-negligible. In addition, these conventional methods generally result in broad particle size distribution, heterogeneous morphology and limited API bioactivity [110,111]. Supercritical fluid technologies are also involved in the scenario of micro- and nanocarrier production. Specifically, two alternative processes can be implemented using SC-CO_2_, namely Supercritical Anti-Solvent (SAS) micronization and Supercritical Assisted Atomization (SAA). These two processes are technological evolutions of the spray drying process [112]. As far as SAS is concerned, the idea is to convey the polymeric solution (comprehensive of the drug) through a nozzle; the solution is thus sprayed inside a high-pressure chamber, previously filled with SC-CO_2_. In this scenario, it is crucial that the solvent be miscible in carbon dioxide, whereas the polymer and the drug are not. Indeed, solution micronization is enhanced by the superimposition of disruptive forces over viscous ones (i.e., droplet average diameter gets smaller), while SC-CO_2_ rapidly extracts the solvent from the fine droplets, leaving only the dry powder to be collected from a filter. An example of a typical SAS configuration is reported in several scientific works [113,114]. From a thermodynamical point of view, the SAS process can also be seen as a different declination of SAPIM, with the exception that the operating point is shifted towards a lower polymer concentration than the one related to the formation of continuous porous structures [88]. Overall, the SAS process is eligible for the co-precipitation of polymer–drug systems not soluble in carbon dioxide. On the other hand, the SAA process is meant to be used when the species exhibit good solubility in carbon dioxide. In this case, the drug/polymer/solvent solution is conveyed to a packed column, where it is put in contact with SC-CO_2_; thus, an expanded liquid is formed and sprayed through a nozzle. This supercritical mixture exhibits near-zero surface tension and low viscosity; thus, the droplets are extremely fine [115,116]. In this process, particle formation takes place in an inert-rich environment, which induces solvent evaporation and solid precipitation. Indeed, SAA, contrary to SAS, can be adopted even when the solvent is water-based, since SC-CO_2_ basically acts as a modifier of fluid-dynamic properties of the original solution. The qualitative representative schemes of SAS and SAA processes are reported in Figure 6.

Numerous examples of supercritical micronization and atomization are reported in the scientific literature. Wang et al. [117] tackled the issue of the low solubility of betamethasone in an aqueous environment, producing PVP-based polyvinyl coprecipitates using the SAS technology. The utilization of SC-CO_2_ allowed drug amorphization, reduced average particle diameter, but also improved bioavailability and cell survival rate. Lu et al. [118] produced hesperetin-PVP (poly(vinylpyrrolidone)) sub-microparticles, optimizing the operating conditions via Box–Behnken design, taking particle size as the response variable. The optimal operating conditions were 100 bar and 40 °C, using 5 mL/min as the solution flow rate, to which corresponds an average diameter of 211 nm. Indeed, the field emission scanning electron microscopy (FESEM) images prove that the particles are spherical, and exhibit a narrow distribution: this kind of observation is appealing for drug delivery. Indeed, the narrower the particle size distribution is, the more precise the release should be. Interestingly enough, the sub-microparticles were able to gradually release the drug in over 48 h, suggesting that polymeric co-precipitate can also be chosen for sustained release purposes. Also, the biological activity was enhanced, suggesting that SAS-based techniques show potential for biomedical applications. Cerro et al. [119] discuss the coprecipitation of β-sitosterol within PCL using SAS. The produced particles were micrometric or submicrometric, although their morphology depends on the operating conditions: it is possible to obtain unimodal spherical particles, irregular clusters or compact fibers, changing the operating pressure. The optimal operating conditions for this system were found to be 90 bar and 40 °C. Also, in vitro drug release tests were carried out and the Ritger–Peppas model effectively described the experimental points, suggesting that higher polymer concentrations result in slower release rates. It stands to reason that if the structure is dense, there is a greater obstacle to overcome. Practically speaking, it is possible that the drug is released faster in the first moments, and then it gradually diffuses from the matrix (taking up to about 120 h). It should be also considered that drug release from polymeric matrices is affected by the competition between erosion, dissolution and diffusion, so there are different mechanisms that weigh in. Vallejo et al. [120] synthesized acetazolamide-recombinamers for glaucoma treatment. They found out that microparticles disintegrate upon contact with physiological fluids, releasing smaller drug nanoparticles in up to 7 h. Moreover, the formulation was proved to relieve intraocular tension and not to generate an inflammatory response: its effectiveness stemmed from the co-existence of hydrophilic and hydrophobic groups, which enhance drug stability and bioavailability. Not unlike the porous structures, polymeric micro-/nanoparticles have multiple purposes: they can be used for sustained and targeted drug release, but also for solubility enhancement and rapid release of lipophilic compounds. For instance, Wang and co-workers [121] developed a coaxial configuration for the supercritical precipitation of curcumin/PVP particles. Also, for this system, the choice of the operating conditions is crucial for defining physio-chemical, thermal, and morphological characteristics. In particular, the high drug-to-polymer ratio interferes with particle formation due to incomplete precipitation, but on the contrary, excess polymer results in increased solution viscosity and the formation of large aggregates; pressure intensifies the disruptive forces, until density increases so much that the diffusion stage is hindered; temperature seems to have a noticeable positive effect on particle morphology, probably due to accelerated solvent evaporation kinetics, until the moment in which thermal transition outbalances correct particle formation. Drug dissolution tests were successful, since the release of curcumin was accelerated and completed in about 5 min. Overall, SAS technology is widely studied from both a theoretical and practical point of view, proving that it is a ready-to-use strategy to generate micro-/nanometric PDCs. However, prior to deciding the most effective process, system thermodynamics should be considered: the SAS process is clearly a powerful tool when both the polymer and the drug are not miscible in SC-CO_2_, but that is not always the case. As pointed out by Iannaco et al. [122], who compared SAS and SAA for the production of PVP-acyclovir, if drug solubility in carbon dioxide is non-negligible, encapsulation efficiency worsens and the overall advantages of supercritical fluid technologies are lost. Although SAS-produced particles were smaller in diameter than the SAA ones, the potential acyclovir extraction during the process has to be considered. In addition, the SAS process requires DMSO (dimethyl sulfoxide), whereas SAA does not (i.e., it requires water/ethanol solutions); another eye-catching aspect is that the CO_2_ volume needed to perform SAS is about 7–8 times larger than that needed for SAA, which is mainly due to the different volumes involved. Indeed, the SAA process requires only a small saturator in which mixing takes place, while SAS needs a whole larger high-pressure chamber to guarantee atomization, solvent extraction and precipitation. Clearly, from an economic perspective, the SAA process seems to be more convenient than SAS, but no general rule should be applied on a first basis: the definition of the operative strategy should rely on thermodynamic parameters rather than only the volumes needed to ultimately complete particle formation, so some aspects should be of primary concern: (i) What is the solubility of the drug in carbon dioxide? (ii) In which solvent does the drug solubilize? (iii) Does a potential drug extraction pay back the investment? Even though there are some aspects of interest when it comes to the choice of the micronization/atomization process, it is also true that the SAA process draws interest, despite the fact that the technology is not as ripe as SAS. For instance, Baldino et al. produced cannabinoid/PVP coprecipitates using the SAA apparatus [123,124]. Since cannabinoids are lipophilic compounds, and hence their solubility in carbon dioxide is high, SAA was adopted; for both cannabidiol (CBD) and cannabigerol (CGB), solubilization rate was improved when compared to the raw powder, suggesting that microparticles are an eligible tool to facilitate cannabinoid delivery in the human body, and to overcome the biological barriers that hinder their absorption. Wu et al. [125] obtained hydroxychloroquine-albumin spherical nanoparticles, with the aim of obtaining gradual drug release. In this case, the plateau was reached in about 1000 min, which is a long time compared to pure drug (i.e., less than 1 h), achieving the task of controlling the release kinetics. Indeed, the denser the biopolymeric network, the longer the time needed to reach the plateau value. Supercritical micronization/atomization processes can also be used to generate inclusion complexes. For instance, Wu et al. [126] produced beclomethasone dipropionate-gamma cyclodextrin composite particles by SAA, obtaining slightly sub-micrometric particles. In this case, drug release was anticipated to improve drug bioavailability.

To sum up, supercritical CO_2_-assisted technologies are flexible tools to generate micro- and nanosized PDCs. Assuming drug solubility and its affinity with the chosen carrier, it is possible to move between SAA and SAS processes. Even though they differ in terms of configuration and fundamental principles, they are both valid candidates for PDC production, improving bioavailability, solubility, stability, and therapeutic effectiveness, proving once again that supercritical fluid processes are versatile and competitive with respect to their conventional counterparts, despite the high costs involved in the development of high-pressure equipment.

## 5. Polymeric Microcapsules

**Figure 7:**
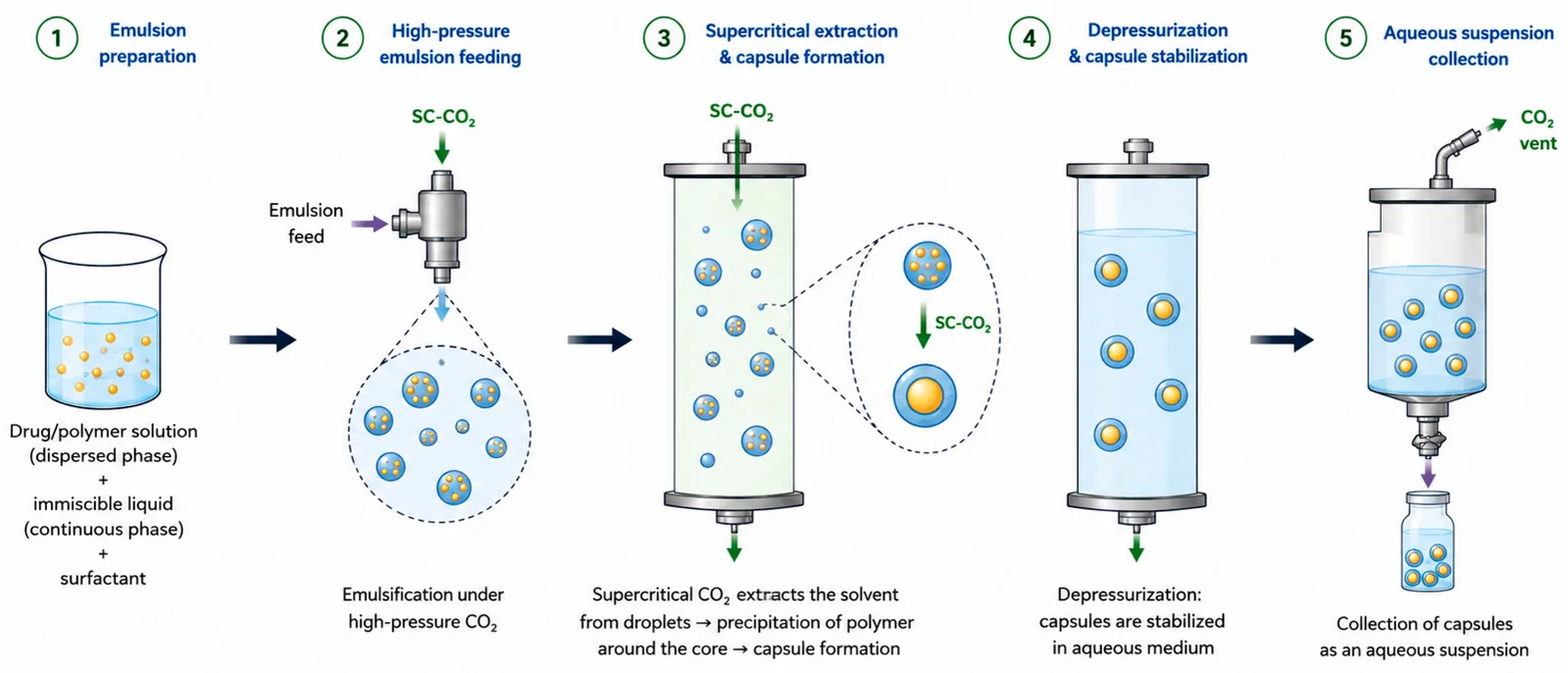
Scheme of SEE process.

Polymeric microcapsules are core–shell particulate systems, consisting of an inner core rich in the API of interest, and of an external polymeric outer shell that actively protects the core and regulates drug diffusion [127,128]. Unlike polymeric micro- and nanoparticles (i.e., microspheres), microcapsules physically confine the active compound inside its core, while the former allow its homogenous dispersion throughout a dense and compact polymeric network. Polymeric microcapsules thus provide enhanced protection against degradation, and allow precise control over drug release and stimuli-responsiveness [129,130,131]. The most widely implemented techniques to obtain polymeric microcapsules include emulsion-based methods, microfluidics, coacervation, interfacial polymerization, and spray drying [129,132,133]. Among these, emulsion-based technologies dominate this panorama thanks to several practical advantages, like broad polymer compatibility (PLGA, PLA, PCL, Eudragit, etc.), possibility to encapsulate both hydrophilic and hydrophobic compounds depending on the emulsion type (O/W, W/O, O/W/O, W/O/W—oil-in-water, water-in-oil, oil-in-water-in-oil, water-in-oil-in-water, respectively), good control over particle characteristics, and relatively straightforward scale-up [127,134,135]. However, just like most conventional techniques, emulsion-based methods also eventually require the utilization of organic solvents and post-processing steps; this drawback can be overcome in this case as well by means of supercritical fluid-based technologies. In this context, Supercritical Emulsion Extraction (SEE) can be employed, whose basic working principle relies on the affinity between the oil phase and SC-CO_2_ and its consequent extraction. The suspension obtained is monodisperse, stable, and features low solvent residues as well as high encapsulation efficiency [136,137]. A scheme of the SEE process is reported in Figure 7.

Nevertheless, it should be highlighted that microfluidics is also an interesting alternative to generate capsules, since it allows exceptionally high control over droplet size distribution [138]. However, it is not devoid of the need to remove the organic solvent from the emulsion, eventually leading to high solvent residue or evaporation-induced consolidation [138,139]. Moreover, microfluidics is effective for generating stable emulsions, but its throughput is limited to single-device systems, inducing the need for further scale-up and device parallelization, in contrast with the higher productivity inherent with supercritical fluid technologies. The true difference lies between encapsulation efficiency and solvent residue: while microfluidics encapsulation efficiency is competitive, its solvent residue is not when compared to SEE [140,141].

For instance, Park [142] explored the effect of process parameters (W/O/W emulsion preparation and SEE) on protein encapsulation in PLGA microcapsules. It was observed that the emulsion preparation stage is crucial to bovine serum albumin (BSA) encapsulation efficiency; namely, primary emulsion homogenization speed sensibly improves encapsulation efficiency. Also, the higher the ratio between the average droplet diameter of the secondary emulsion, the better the encapsulation efficiency. However, the maximum EE% recorded was about 50%, indicating space for optimization. Moreover, long homogenization periods worsen encapsulation efficiency (EE%), probably due to drug diffusion from the droplets to the bulk. As far as SEE operating parameters are concerned, when pressure increases, diffusion coefficients are enhanced and the drug migrates out of the capsules, which is also facilitated by the plasticization effect of SC-CO_2_, lowering the overall EE%. On the other hand, temperature does not have a linear effect of EE%, and it is not dissociated from pressure. Indeed, fluid-dynamic equilibria are clearly non-ideal in the supercritical domain, meaning that there should always be a trade-off between CO_2_ solvent power, diffusivity coefficients, and species solubility. The most efficient encapsulation (67.75%) was registered at 85 bar and 35 °C. Liu et al. [143] proposed a SEE configuration that involves emulsion spraying rather than contacting in a packed column. They observed the interfacial changes between the solvent (ethyl acetate) and SC-CO_2_ as a function of pressure and temperature, reaching conclusions not unlike the ones highlighted during the discussion of the SAS process; namely, increasing pressure enhances jet breakup and induces vanishing of the phase interface, due to the weakening of viscous resistances, while temperature can influence system viscosity and induce a reduction in solvent droplet size. These phenomena were also explored in an attempt to encapsulate an antitumor drug (i.e., paclitaxel) in PLGA-based emulsions. The optimal operating point was observed at 90 bar and 40 °C, to which corresponds an EE% of 89.7%, confirming the high potential of SEE in achieving high performances. Also, the solvent residue was lower than 30 mg/L, considerably smaller than other plant configurations (bubble, spray or countercurrent columns). Interestingly, the drug release was sustained over a period of up to 50 h, suggesting that it is governed by pure Fickian diffusion. These dynamics can be attributed to the fact that the polymeric outer shell is extremely thin, while the core is liquid: there are no porosities or interconnected cavities. However, it is also important to underline that there is no general rule that dictates the release rate from capsules or spheres: even though micro- and nanoparticles are complex, dense systems, it is possible that the drug release could be anticipated when compared to microcapsules, depending on the structure and chemical composition. For instance, Iannaco et al. [144] also prepared PLGA-based micro- and nanocapsules, loaded with bromelain: the estimated value of EE% was about 94% after a double ultrasonication step during emulsion preparation, which was considerably high. Moreover, bromelain was released in 50 h, but they suggest that only the first steps of the process are governed by diffusion, while the latter parts are dictated by PLGA hydrolysis. On the contrary, Vizcaya et al. [145] used Eudragit^®^ S100 as a biopolymeric shell and loaded the capsules with 5-aminosalicylic acid. Not only did they obtain nanometric capsules (165 nm), but also high EE% (84%). The choice of Eudragit^®^ S100 as a biopolymeric shell allowed pH-responsiveness, inducing rapid release of the drug in the stomach environment (>80%) and the rest in the intestinal trait, justifying a stomach-targeted treatment. To produce these capsules, DMSO was used: despite its higher boiling point, SEE allows abatement of its content in the final formulation down to 4% (computed via sulfur content estimation), while solvent evaporation was reduced to just 21%, showing that supercritical fluids substantially help mitigate toxicity. The authors also evaluated the possibility of using dichloromethane or tetrahydrofuran for capsule production, but chose to work with DMSO despite its high boiling point due to its affinity with the drug and good solubility in carbon dioxide. Park et al. [146] prepared PLGA-exenatide capsules, by means of SEE and of solvent evaporation. They observed that the capsules produced by supercritical emulsion extraction exhibited improved properties, including higher EE% (82.9 ± 2.1% vs. 73.6 ± 2.7%), lower residual solvent (85.4 ± 2.1 ppm vs. 627.5 ± 6.1 ppm) and reduced particle size. Moreover, they showed promising in vivo results, and in vitro drug release tests showed that the drug was released sustainably in 24 days, featuring a burst release about half that of capsules produced via solvent evaporation (8.5 ± 0.7% vs. 13.9 ± 1.4%). Overall, it can be stated that the improvements in final properties are due to supercritical fluid processing.

To sum up, polymeric capsules are also interesting candidates for drug delivery applications. In light of the considerations carried out during the present discussion, it emerges that SAS and SAA allow for better particle size control, specifically depending on operating conditions; nanocapsule size instead is much more dependent on emulsion preparation, although SEE enables morphology preservation and narrower particle size distributions when compared to conventional production strategies. When available, solvent residue measurements confirm that supercritical fluid technologies lead to negligible solvent content, and the prolonged drug release suggests proper encapsulation and stability over time. As a general rule, it seems that capsules lead to sustained drug release over wider time windows than SAS or SAA, but the outcome also depends on polymer concentration and polymer–drug combination. Still, a comparative analysis of energy requirements should be carried out, in order to put other parameters on the table to allow comprehensive decision making on PDC production. Table 2 synthesizes the discussed papers on micro- and nanocarrier production, their method and the most relevant conclusions.

## 6. Conclusions

Supercritical fluid technologies are powerful tools to obtain polymeric drug carriers. Indeed, there are numerous possibilities to choose from, depending on the desired application. In this work, several polymeric drug carriers were analyzed, ranging from 3-D porous structures to micro- and nanoparticles and nanocapsules, focusing attention on bio-based systems (e.g., chitosan, alginate, pectin, PCL, PVP, PLGA, etc.). Each biopolymer is suitable for the generation of a specific drug carrier, but some of them are versatile and can be used for multiple purposes. For instance, chitosan, alginate or pectin can be declined for both porous structure and particle production, while polymers like PMMA are mostly viable for nanocapsule production due to their physico-chemical characteristics. However, it should be noted that some requirements should be met. For example, for SGD, polymer gelling ability must be provided; for SAS and SAPIM, the polymer should be immiscible in CO_2_, while the solvent must be miscible; in the case of supercritical foaming, the polymer must undergo transition to the glassy regime in the operating window. So, basically, the drug + polymer thermodynamics dictates the best approach in terms of processability.xc

However, a question may arise: What kind of PDC is to be chosen among this wide range of possibilities? As a general rule, porous structures can be used for topical applications, regenerative medicine, but also oral drug delivery (for both sustained and anticipated drug release); micro- and nanoparticles can be chosen for oral drug delivery, considering that the release timeframe could go from a few minutes to some days; finally, capsules can be used when the application is target-specific or when the release should last from days to weeks. This review proves that supercritical fluids are effective and versatile tools for generating polymeric drug carriers, paving the way for the advancement of innovative systems in the pharmaceutical field. It should be acknowledged that supercritical fluid technologies are being explored mainly on a lab scale rather than full plant scale, although some directions are more well-paved than others. Porous structures are—at present—more industrially ready than particles and capsules, proof of that being that their production in some cases is at pilot plant scale. Thus, among the proposed technologies, supercritical gel drying and foaming sit at the forefront of industrial translation, while SAPIM, SAA, SAS, and SAA are in an explorative phase that precedes larger-scale production. The full development of supercritical fluid technologies is—at present—hindered by the non-negligible cost of high-pressure equipment, but given the higher quality of the final products, they are worth further exploration. Future directions should include economic, environmental, and sustainability evaluations to be complemented by technical studies, to overview the plant-scale feasibility of such processes.

## Figures and Tables

**Table 1:** Literature survey summary of aerogel-based PDCs.

Aerogel/API	Drug Loading Method	Application	Notes	Ref.
Chitosan microparticles/cloripramine	Post-drying wet impregnation	Nasal drug delivery	Aerogel microparticles allow rapid and selective release in the hippocampus (30 min)	[75]
Egg white protein microparticles/cloripramine	Post-drying wet impregnation	Nasal drug delivery	Cloripramine concentration spiked in the hippocampus in 10 min	[76]
Citrus pectin microparticles/vanillin	Supercritical fluid impregnation	Oral drug delivery	Vanillin release is affected by textural properties; plateau is reached in maximum 60 min	[77]
Alginate-based microparticles/ibuprofen	Supercritical fluid impregnation	Oral drug delivery	High drug loading efficiency (93.5%); rapid drug release (>90% in 15 min)	[37]
Pectin beads/ibuprofen	Supercritical fluid impregnation (ethanol as co-solvent)	Oral drug delivery	Lower drug loading efficiency (60.0%); drug loading in aerogels is higher than in cryogels; drug release is retarded (3 h)	[78]
Silk fibroin-alginate beads/ciprofloxacin	Pre-gelation	Hemostatic active devices	The drug is released in 24 h; the aerogel is stable after days of utilization	[79]
Alginate-konjac glucomannan core–shell beads/ibuprofen-dexamethasone base	Pre-gelation	Oral drug delivery	Aerogels accelerate the release of poorly soluble drugs, and delay the release of soluble compounds	[80]
Tragacanth-alginate monoliths/ibuprofen	Post-drying wet impregnation	Oral drug delivery	Drug release is pH-responsive; longer times needed to reach plateau (400 min)	[81]
Starch monoliths/quercetin	Supercritical fluid impregnation	Fungistatic agents, wound healing/dressing	Release rate depends on textural properties; plateau reached in 140 min	[82]
Carboxymethyl cellulose monoliths/L-ascorbic acid 2-phosphate	Pre-gelation	Oral drug delivery	Diffusion coefficients are low due to the nanostructure; aerogels are eligible for controlled-rate purposes	[83]
Tragacanth-alginate monoliths/paracetamol	Solvent exchange	Oral drug delivery	Drug release can be sustained over 6–7 h	[84]

**Table 2:** Literature survey summary of micro-/nanocarriers.

Drug Delivery Device	Supercritical Technology	Notes	Refs.
PVP/betamethasone microspheres	SAS	Enhanced bioavailability and cell survival rate	[117]
PVP/hesperetin nanospheres	SAS	Particle size distribution was unimodal and narrow (211 nm); hesperetin drug release was sustained over 48 h	[118]
PCL/β-sitosterol microspheres	SAS	Higher network density slows down the release; burst release is followed by diffusion in 120 h	[119]
Recombinamers/acetazolamide microspheres	SAS	Drug nanoparticles are dispersed inside microspheres, allowing for delayed drug release	[120]
PVP/curcumin microspheres	SAS	Curcumin solubility was improved; drug release was immediate	[121]
PVP/acyclovir micro- and nanospheres	SAA/SAS	SAA avoids drug extraction; SAS enhances particle reduction; drug release profile is accelerated (10–20 min)	[122]
PVP/cannabinoids nanoparticles	SAA	Cannabinoid solubility was enhanced; drug release and absorption were facilitated	[123,124]
Albumin/hydroxychloroquine nanoparticles	SAA	Drug dissolution was delayed due to the dense polymeric network; the plateau was reached after 1000 min	[125]
Beclomethasone dipropionate/β-cyclodextrin inclusion complex nanoparticles	SAA	Drug release and bioavailability are enhanced	[126]
BSA/PLGA nanocapsules	SEE	Both emulsion preparation and SEE processing parameters influence EE%; 67.75% of the drug was encapsulated	[142]
Paclitaxel/PLGA nanocapsules	SEE	Emulsion spray drying follows the atomization principles; EE% depends on the extraction configuration	[143]
Bromelain/PLGA nanocapsules	SEE	Maximum EE% was 94%; drug release is prolonged to 50 h and governed by diffusion	[144]
5-aminosalicylic acid/Eudragit^®^ S100 nanocapsules	SEE	The polymeric shell can confer pH-responsive attributes; the drug can be released precisely in the stomach	[145]
Exenatide/PLGA nanocapsules	SEE	SEE results in improved biological activity when compared to solvent evaporation; drug release was sustained for 24 days	[146]

## Data Availability

No new data were created or analyzed in this study. Data sharing is not applicable to this article.

## Abbreviations

The following abbreviations are used in this manuscript:

API	Active Pharmaceutical Ingredient
BSA	Bovine Serum Albumin
CBD	Cannabidiol
CBG	Cannabigerol
DDS	Drug Delivery System
DMSO	Dimethyl sulfoxide
EE%	Encapsulation Efficiency (%)
FESEM	Field Emission Scanning Electron Microscopy
NIPS	Non-Solvent-Induced Phase Separation
O/W	Oil-in-Water
O/W/O	Oil-in-Water-in-Oil
PBAT	Polybutylene Adipate Terephthalate
PBS	Phosphate Buffered Saline
PCL	Poly(ε-caprolactone)
PDC	Polymeric Drug Carrier
PEG	Poly(ethylene glycol)
PLA	Poly(lactic acid)
PLGA	Poly(lactic-co-glycolic acid)
PVP	Poly(vinylpyrrolidone)
SAA	Supercritical Assisted Atomization
SAPIM	Supercritical Assisted Phase Inversion Method
SAS	Supercritical AntiSolvent
SC-CO_2_	Supercritical carbon dioxide
SEE	Supercritical Emulsion Extraction
SFI	Supercritical Fluid Impregnation
SGD	Supercritical Gel Drying
SSA	Specific Surface Area
TIPS	Thermally Induced Phase Separation
VOCs	Volatile Organic Compounds
W/O	Water-in-Oil
W/O/W	Water-in-Oil-in-Water

## References

[1] Sultana A., Zare M., Thomas V., Kumar T.S.S., Ramakrishna S. (2022). Nano-Based Drug Delivery Systems: Conventional Drug Delivery Routes, Recent Developments and Future Prospects. Med. Drug Discov..

[2] Li C., Wang J., Wang Y., Gao H., Wei G., Huang Y., Yu H., Gan Y., Wang Y., Mei L. (2019). Recent Progress in Drug Delivery. Acta Pharm. Sin. B.

[3] Yu Y., Gan C., Shi K., Bei Z., Yu Y., Pan M., Deng H., Qian Z. (2026). Recent Advances in Drug Delivery Systems for Pulmonary Fibrosis Therapy. Chin. Chem. Lett..

[4] Swain S., Pratap Singh A., Yadav R.K. (2022). A Review on Polymer Hydrogel and Polymer Microneedle Based Transdermal Drug Delivery System. Mater. Today Proc..

[5] Beach M.A., Nayanathara U., Gao Y., Zhang C., Xiong Y., Wang Y., Such G.K. (2024). Polymeric Nanoparticles for Drug Delivery. Chem. Rev..

[6] Jassem N.A. (2022). Orodispersible Tablets: A Review on Recent Trends in Drug Delivery. Int. J. Drug Deliv. Technol..

[7] Hoffmann S.V., O’Shea J.P., Galvin P., Jannin V., Griffin B.T. (2024). State-of-the-Art and Future Perspectives in Ingestible Remotely Controlled Smart Capsules for Drug Delivery: A GENEGUT Review. Eur. J. Pharm. Sci..

[8] Ke W.-R., Chang R.Y.K., Chan H.-K. (2022). Engineering the Right Formulation for Enhanced Drug Delivery. Adv. Drug Deliv. Rev..

[9] Hamdy A., El-Badry M., Fathy M., El-Sayed A.M. (2024). Impact of Oil Type on the Development and Oral Bioavailability of Self-Nanoemulsifying Drug Delivery Systems Containing Simvastatin. Sci. Rep..

[10] Matharoo N.S., Garimella H.T., German C., Przekwas A.J., Michniak-Kohn B. (2023). A Comparative Evaluation of Desoximetasone Cream and Ointment Formulations Using Experiments and In Silico Modeling. Int. J. Mol. Sci..

[11] Wang Q., Atluri K., Tiwari A.K., Babu R.J. (2023). Exploring the Application of Micellar Drug Delivery Systems in Cancer Nanomedicine. Pharmaceuticals.

[12] Xu L., Wang X., Liu Y., Yang G., Falconer R.J., Zhao C.-X. (2022). Lipid Nanoparticles for Drug Delivery. Adv. NanoBiomed Res..

[13] Asad S., Jacobsen A.-C., Teleki A. (2022). Inorganic Nanoparticles for Oral Drug Delivery: Opportunities, Barriers, and Future Perspectives. Curr. Opin. Chem. Eng..

[14] Wang J., Li B., Qiu L., Qiao X., Yang H. (2022). Dendrimer-Based Drug Delivery Systems: History, Challenges, and Latest Developments. J. Biol. Eng..

[15] Parvin N., Kumar V., Joo S.W., Mandal T.K. (2024). Emerging Trends in Nanomedicine: Carbon-Based Nanomaterials for Healthcare. Nanomaterials.

[16] Raina N., Rani R., Thakur V.K., Gupta M. (2023). New Insights in Topical Drug Delivery for Skin Disorders: From a Nanotechnological Perspective. ACS Omega.

[17] Hua S. (2020). Advances in Oral Drug Delivery for Regional Targeting in the Gastrointestinal Tract—Influence of Physiological, Pathophysiological and Pharmaceutical Factors. Front. Pharmacol..

[18] Lou J., Duan H., Qin Q., Teng Z., Gan F., Zhou X., Zhou X. (2023). Advances in Oral Drug Delivery Systems: Challenges and Opportunities. Pharmaceutics.

[19] Adepu S., Ramakrishna S. (2021). Controlled Drug Delivery Systems: Current Status and Future Directions. Molecules.

[20] Abbasi R., Shineh G., Mobaraki M., Doughty S., Tayebi L. (2023). Structural Parameters of Nanoparticles Affecting Their Toxicity for Biomedical Applications: A Review. J. Nanopart Res..

[21] Eker F., Duman H., Akdaşçi E., Bolat E., Sarıtaş S., Karav S., Witkowska A.M. (2024). A Comprehensive Review of Nanoparticles: From Classification to Application and Toxicity. Molecules.

[22] Guidi L., Cascone M.G., Rosellini E. (2024). Light-Responsive Polymeric Nanoparticles for Retinal Drug Delivery: Design Cues, Challenges and Future Perspectives. Heliyon.

[23] Bhairam M., Shukla S.S., Pandey R.K., Gidwani B. (2026). Emerging Frontiers of Responsive Nanomaterials in Drug Delivery Systems: From Design to Clinical Translation. OpenNano.

[24] Yazdani S., Mozaffarian M., Pazuki G., Hadidi N., Villate-Beitia I., Zárate J., Puras G., Pedraz J.L. (2024). Carbon-Based Nanostructures as Emerging Materials for Gene Delivery Applications. Pharmaceutics.

[25] Thakor P., Bhavana V., Sharma R., Srivastava S., Singh S.B., Mehra N.K. (2020). Polymer–Drug Conjugates: Recent Advances and Future Perspectives. Drug Discov. Today.

[26] Liu M., Zhong L., Yan T., Jiang Z., Zhou L. (2026). Bacterial Microenvironment-Responsive Polymeric Carriers for Antibacterial Agent Delivery. Biomacromolecules.

[27] Pratap Singh L., Patro L.R., Sayeed M., Rani Mandadi S., Chandra Pant N., Panda C., Aparna T.N., Vodeti R., Das C. (2025). Polymeric Drug Delivery Systems: Chemical Design Functionalization and Biomedical Applications. J. Chem. Rev..

[28] Mu X., Liu Y., Zhang J., Yin Y., Diao A., Jiao Y., Chen S., Xu D., Li G., Zheng Z. (2026). All-Silk Fibroin–Based Dual-Drug Delivery System for Antibacterial and Antioxidant Wound Repair. Int. J. Biol. Macromol..

[29] Procopio A., Lagreca E., Jamaledin R., La Manna S., Corrado B., Di Natale C., Onesto V. (2022). Recent Fabrication Methods to Produce Polymer-Based Drug Delivery Matrices (Experimental and In Silico Approaches). Pharmaceutics.

[30] Virameteekul S., Lees A.J., Bhidayasiri R. (2024). Small Particles, Big Potential: Polymeric Nanoparticles for Drug Delivery in Parkinson’s Disease. Mov. Disord..

[31] Avramović N., Mandić B., Savić-Radojević A., Simić T. (2020). Polymeric Nanocarriers of Drug Delivery Systems in Cancer Therapy. Pharmaceutics.

[32] Xia W., Tao Z., Zhu B., Zhang W., Liu C., Chen S., Song M. (2021). Targeted Delivery of Drugs and Genes Using Polymer Nanocarriers for Cancer Therapy. Int. J. Mol. Sci..

[33] Benalaya I., Alves G., Lopes J., Silva L.R. (2024). A Review of Natural Polysaccharides: Sources, Characteristics, Properties, Food, and Pharmaceutical Applications. Int. J. Mol. Sci..

[34] Mahmud M.Z.A., Islam M.D., Mobarak M.H. (2023). The Development of Eco-Friendly Biopolymers for Use in Tissue Engineering and Drug Delivery. J. Nanomater..

[35] Lai J., Azad A.K., Sulaiman W.M.A.W., Kumarasamy V., Subramaniyan V., Alshehade S.A. (2024). Alginate-Based Encapsulation Fabrication Technique for Drug Delivery: An Updated Review of Particle Type, Formulation Technique, Pharmaceutical Ingredient, and Targeted Delivery System. Pharmaceutics.

[36] Alkhalidi H.M., Alahmadi A.A., Rizg W.Y., Yahya E.B., Abdul Khalil H.P.S., Mushtaq R.Y., Badr M.Y., Safhi A.Y., Hosny K.M. (2024). Revolutionizing Cancer Treatment: Biopolymer-Based Aerogels as Smart Platforms for Targeted Drug Delivery. Macromol. Rapid Commun..

[37] Alnaief M., Mohammad B., Altarawneh I., Alkhatib D., Al-Hamamre Z., Mashaqbeh H., Bani-Melhem K., Obeidat R. (2025). Hybrid Alginate-Based Polysaccharide Aerogels Microparticles for Drug Delivery: Preparation, Characterization, and Performance Evaluation. Gels.

[38] Kim Y., Kwak J., Lim M., Lim S.Y., Chae S., Ha S.-J., Won Y.-W., Kim H.-O., Lim K.S. (2025). Advances in PCL, PLA, and PLGA-Based Technologies for Anticancer Drug Delivery. Pharmaceutics.

[39] Rajendiran S., Deivanayagam P. (2025). Synthetic Biopolymers–Assisted Polycaprolactone Blends for Pharmacology: A Comprehensive Review With Recent Insights. Polym. Adv. Techs.

[40] Chen X., Wu Y., Dau V.T., Nguyen N.-T., Ta H.T. (2023). Polymeric Nanomaterial Strategies to Encapsulate and Deliver Biological Drugs: Points to Consider between Methods. Biomater. Sci..

[41] Li X., Li L., Wang D., Zhang J., Yi K., Su Y., Luo J., Deng X., Deng F. (2024). Fabrication of Polymeric Microspheres for Biomedical Applications. Mater. Horiz..

[42] Zhang J., Guo M., Luo M., Cai T. (2023). Advances in the Development of Amorphous Solid Dispersions: The Role of Polymeric Carriers. Asian J. Pharm. Sci..

[43] Lobel B.T., Baiocco D., Al-Sharabi M., Routh A.F., Zhang Z., Cayre O.J. (2024). Current Challenges in Microcapsule Designs and Microencapsulation Processes: A Review. ACS Appl. Mater. Interfaces.

[44] Tabernero A., González-Garcinuño Á., Cardea S., Martín Del Valle E. (2022). Supercritical Carbon Dioxide and Biomedicine: Opening the Doors towards Biocompatibility. Chem. Eng. J..

[45] Singh N., Vinjamur M., Mukhopadhyay M. (2022). Influence of Drug Properties on Loadings and Release Kinetics of Drugs from Silica Aerogels Loaded in Supercritical CO_2_. J. Supercrit. Fluids.

[46] Alamoudi J.A. (2024). Recent Advancements toward the Incremsent of Drug Solubility Using Environmentally-Friendly Supercritical CO_2_: A Machine Learning Perspective. Front. Med..

[47] Gallo M., Onida B., Banchero M. (2024). Supercritical Techniques for Pharmaceutical Applications. Pharmaceutics.

[48] Shi J., Xue S., Sun Q., Scanlon M. (2024). Effects of Solubility of Supercritical-CO_2_ Solvent and Mass Transfer Property on Extraction of Vitamin E from Canola Seeds. LWT.

[49] Hazaveie S.M., Sodeifian G., Saadati Ardestani N. (2024). Micro and Nanosizing of Tamsulosin Drug via Supercritical CO_2_ Antisolvent (SAS) Process. J. CO2 Util..

[50] Bento C.S.A., Alarico S., Empadinhas N., De Sousa H.C., Braga M.E.M. (2022). Sequential scCO_2_ Drying and Sterilisation of Alginate-Gelatine Aerogels for Biomedical Applications. J. Supercrit. Fluids.

[51] Chakravarty P., Famili A., Nagapudi K., Al-Sayah M.A. (2019). Using Supercritical Fluid Technology as a Green Alternative During the Preparation of Drug Delivery Systems. Pharmaceutics.

[52] Kankala R.K., Zhang Y.S., Wang S., Lee C., Chen A. (2017). Supercritical Fluid Technology: An Emphasis on Drug Delivery and Related Biomedical Applications. Adv. Healthc. Mater..

[53] Davies O.R., Lewis A.L., Whitaker M.J., Tai H., Shakesheff K.M., Howdle S.M. (2008). Applications of Supercritical CO_2_ in the Fabrication of Polymer Systems for Drug Delivery and Tissue Engineering. Adv. Drug Deliv. Rev..

[54] Aegerter M.A., Leventis N., Koebel M., Steiner Iii S.A. (2023). Springer Handbook of Aerogels.

[55] Payanda Konuk O., Alsuhile A.A.A.M., Yousefzadeh H., Ulker Z., Bozbag S.E., García-González C.A., Smirnova I., Erkey C. (2023). The Effect of Synthesis Conditions and Process Parameters on Aerogel Properties. Front. Chem..

[56] Niculescu A.-G., Tudorache D.-I., Bocioagă M., Mihaiescu D.E., Hadibarata T., Grumezescu A.M. (2024). An Updated Overview of Silica Aerogel-Based Nanomaterials. Nanomaterials.

[57] Walker R.C., Hyer A.P., Guo H., Ferri J.K. (2023). Silica Aerogel Synthesis/Process–Property Predictions by Machine Learning. Chem. Mater..

[58] García-González C.A., Sosnik A., Kalmár J., De Marco I., Erkey C., Concheiro A., Alvarez-Lorenzo C. (2021). Aerogels in Drug Delivery: From Design to Application. J. Control. Release.

[59] Dhua S., Gupta A.K., Mishra P. (2022). Aerogel: Functional Emerging Material for Potential Application in Food: A Review. Food Bioprocess Technol..

[60] Sharma J., Sheikh J., Behera B.K. (2023). Aerogel Composites and Blankets with Embedded Fibrous Material by Ambient Drying: Reviewing Their Production, Characteristics, and Potential Applications. Dry. Technol..

[61] Zhang S., Fan Q., Pan Y., Wang W., Lin R., Chen G. (2024). Role of Freeze-Drying in Cellulose-Based Aerogels: Freezing Optimization Strategies and Diverse Water Treatment Applications. Dry. Technol..

[62] Alvarez-Valcarce J., Mottola S., González-Garcinuño Á., Tabernero A., Misol A., Jiménez A., Cardea S., De Marco I., Martín Del Valle E.M. (2025). Lignin-Kappa-Carrageenan Cryogels and Aerogels: Structure and Swelling Analysis. Int. J. Biol. Macromol..

[63] Basak S., Singhal R.S. (2023). The Potential of Supercritical Drying as a “Green” Method for the Production of Food-Grade Bioaerogels: A Comprehensive Critical Review. Food Hydrocoll..

[64] Demina T.S., Minaev N.V., Akopova T.A. (2024). Polysaccharide-Based Aerogels Fabricated via Supercritical Fluid Drying: A Systematic Review. Polym. Bull..

[65] Illanes-Bordomás C., Landin M., García-González C.A. (2023). Aerogels as Carriers for Oral Administration of Drugs: An Approach towards Colonic Delivery. Pharmaceutics.

[66] Zhang Q., Li L., Wu H., Cheng Y., Liu C., Fang C. (2025). Recent Advances in Cellulose Based Aerogels with Various Dimensions: Design, Functionalization, and Applications. Cellulose.

[67] Bento C.S.A., Ruivo J.P., Alarico S., Empadinhas N., Candeias E., Cardoso S., De Sousa H.C., Dias A.M.A., Braga M.E.M. (2025). Eco-Friendly Development of Sterile Chitosan-Based Aerogels for Wound Dressing Applications Using Integrated scCO_2_ Processing. Int. J. Pharm..

[68] Raman S.P., Keil C., Dieringer P., Hübner C., Bueno A., Gurikov P., Nissen J., Holtkamp M., Karst U., Haase H. (2019). Alginate Aerogels Carrying Calcium, Zinc and Silver Cations for Wound Care: Fabrication and Metal Detection. J. Supercrit. Fluids.

[69] Athamneh T., Hajnal A., Al-Najjar M.A.A., Alshweiat A., Obaidat R., Awad A.A., Al-Alwany R., Keitel J., Wu D., Kieserling H. (2023). In Vivo Tests of a Novel Wound Dressing Based on Agar Aerogel. Int. J. Biol. Macromol..

[70] Veres P., Sebők D., Dékány I., Gurikov P., Smirnova I., Fábián I., Kalmár J. (2018). A Redox Strategy to Tailor the Release Properties of Fe(III)-Alginate Aerogels for Oral Drug Delivery. Carbohydr. Polym..

[71] Del Gaudio P., Auriemma G., Mencherini T., Porta G.D., Reverchon E., Aquino R.P. (2013). Design of Alginate-Based Aerogel for Nonsteroidal Anti-Inflammatory Drugs Controlled Delivery Systems Using Prilling and Supercritical-Assisted Drying. J. Pharm. Sci..

[72] Ozesme Taylan G., Illanes-Bordomás C., Guven O., Erkan E., Erünsal S.Ç., Oztop M.H., García-González C.A. (2025). Core-Shell Aerogel Design for Enhanced Oral Insulin Delivery. Int. J. Pharm..

[73] Boruah M., Sarma A. (2025). Dry Gels: Concept, Current Trends, and New Avenues in Drug Delivery and Biomedical Application. Adv. Healthc. Mater..

[74] García-González C.A., Jin M., Gerth J., Alvarez-Lorenzo C., Smirnova I. (2015). Polysaccharide-Based Aerogel Microspheres for Oral Drug Delivery. Carbohydr. Polym..

[75] Menshutina N., Majouga A., Uvarova A., Lovskaya D., Tsygankov P., Mochalova M., Abramova O., Ushakova V., Morozova A., Silantyev A. (2022). Chitosan Aerogel Particles as Nasal Drug Delivery Systems. Gels.

[76] Lovskaya D., Bezchasnyuk A., Mochalova M., Tsygankov P., Lebedev A., Zorkina Y., Zubkov E., Ochneva A., Gurina O., Silantyev A. (2022). Preparation of Protein Aerogel Particles for the Development of Innovative Drug Delivery Systems. Gels.

[77] Méndez D.A., Schroeter B., Martínez-Abad A., Fabra M.J., Gurikov P., López-Rubio A. (2023). Pectin-Based Aerogel Particles for Drug Delivery: Effect of Pectin Composition on Aerogel Structure and Release Properties. Carbohydr. Polym..

[78] Suttiruengwong S., Konthong S., Pivsa-Art S., Plukchaihan P., Meesuwan P., Wanthong M., Panpradist N., Kurien R.A., Pakawanit P., Sriamornsak P. (2024). Fabrication and Characterization of Porous Pectin-Based Aerogels for Drug Delivery. Carbohydr. Polym. Technol. Appl..

[79] Sellitto M.R., Larobina D., De Soricellis C., Amante C., Falcone G., Russo P., Bernardes B.G., Oliveira A.L., Del Gaudio P. (2025). Silk Fibroin–Alginate Aerogel Beads Produced by Supercritical CO_2_ Drying: A Dual-Function Conformable and Haemostatic Dressing. Gels.

[80] Illanes-Bordomás C., Landin M., García-González C.A. (2025). Novel Core–Shell Aerogel Formulation for Drug Delivery Based on Alginate and Konjac Glucomannan: Rational Design Using Artificial Intelligence Tools. Polymers.

[81] Behruzi M., Moghaddas J., Ramezanian S., Mohammadi A. (2026). Atmospheric Pressure Drying Starch Aerogel as a Drug Carrier for Enhancing Ibuprofen Water Solubility. Biofuels Bioprod. Biorefining.

[82] Mottola S., Iannone G., Giordano M., González-Garcinuño Á., Jiménez A., Tabernero A., Martín Del Valle E., De Marco I. (2023). Supercritical Impregnation of Starch Aerogels with Quercetin: Fungistatic Effect and Release Modelling with a Compartmental Model. Int. J. Biol. Macromol..

[83] Yu S., Budtova T. (2024). Creating and Exploring Carboxymethyl Cellulose Aerogels as Drug Delivery Devices. Carbohydr. Polym..

[84] Al-barudi A., Sinani G., Ulker Z. (2024). Biodegradable Polysaccharide Aerogels Based on Tragacanth and Alginate as Novel Drug Delivery Systems. J. Sol.-Gel Sci. Technol..

[85] Galiano F., Briceño K., Marino T., Molino A., Christensen K.V., Figoli A. (2018). Advances in Biopolymer-Based Membrane Preparation and Applications. J. Membr. Sci..

[86] Guillen G.R., Pan Y., Li M., Hoek E.M.V. (2011). Preparation and Characterization of Membranes Formed by Nonsolvent Induced Phase Separation: A Review. Ind. Eng. Chem. Res..

[87] Cardea S., Baldino L., Reverchon E. (2018). Comparative Study of PVDF-HFP-Curcumin Porous Structures Produced by Supercritical Assisted Processes. J. Supercrit. Fluids.

[88] Cardea S., De Marco I. (2020). Cellulose Acetate and Supercritical Carbon Dioxide: Membranes, Nanoparticles, Microparticles and Nanostructured Filaments. Polymers.

[89] Baldino L., Cardea S., Reverchon E. (2017). Biodegradable Membranes Loaded with Curcumin to Be Used as Engineered Independent Devices in Active Packaging. J. Taiwan Inst. Chem. Eng..

[90] Lucia B., Alvaro G.-G., Antonio T., Stefano C., Del Valle Eva M., Ernesto R. (2023). Production of Cellulose Acetate Membranes Loaded with Quercetin by Supercritical CO_2_ Phase Inversion. Chem. Eng. Trans..

[91] Baldino L., González-Garcinuño Á., Tabernero A., Cardea S., Martín Del Valle E.M., Reverchon E. (2021). Production of Fungistatic Porous Structures of Cellulose Acetate Loaded with Quercetin, Using Supercritical CO_2_. J. Supercrit. Fluids.

[92] Reverchon E., Adami R., Cardea S., Porta G.D. (2009). Supercritical Fluids Processing of Polymers for Pharmaceutical and Medical Applications. J. Supercrit. Fluids.

[93] Tomin M., Kmetty Á. (2022). Polymer Foams as Advanced Energy Absorbing Materials for Sports Applications—A Review. J. Appl. Polym. Sci..

[94] Jin F.-L., Zhao M., Park M., Park S.-J. (2019). Recent Trends of Foaming in Polymer Processing: A Review. Polymers.

[95] Reglero Ruiz J.A., Vincent M., Agassant J., Sadik T., Pillon C., Carrot C. (2015). Polymer Foaming with Chemical Blowing Agents: Experiment and Modeling. Polym. Eng. Sci..

[96] Seo J.-H., Han J., Lee K.S., Cha S.W. (2012). Combined Effects of Chemical and Microcellular Foaming on Foaming Characteristics of PLA (Poly Lactic Acid) in Injection Molding Process. Polym.-Plast. Technol. Eng..

[97] Zhai W., Jiang J., Park C.B. (2022). A Review on Physical Foaming of Thermoplastic and Vulcanized Elastomers. Polym. Rev..

[98] Chen B., Jiang J., Wang Z., Li Y., Tian F., Wang L., Zhai W. (2024). Controlling the Crystal Morphology of High-Hardness TPU through Two Pre-Crystallization Processes and Its Impact on Physical Foaming Behavior. Polymer.

[99] Jacobs L.J.M., Kemmere M.F., Keurentjes J.T.F. (2008). Sustainable Polymer Foaming Using High Pressure Carbon Dioxide: A Review on Fundamentals, Processes and Applications. Green Chem..

[100] Dong M., Wang G., Zhang X., Tan D., Ren J., Colorado H., Hou H., Toktarbay Z., Guo Z. (2023). An Overview of Polymer Foaming Assisted by Supercritical Fluid. Adv. Compos. Hybrid Mater..

[101] Tomasko D.L., Burley A., Feng L., Yeh S.-K., Miyazono K., Nirmal-Kumar S., Kusaka I., Koelling K. (2009). Development of CO_2_ for Polymer Foam Applications. J. Supercrit. Fluids.

[102] Hu X., Luo X., Wang G., Dong M., Zhou L., Pan X., Du M., Zhang X., Li K., Zhang X. (2026). Conductive Polymer Foaming: A Review on Fundamentals, Technology and Applications. Polymers.

[103] Satpayeva A., Rojas A., Tyrka M., Ksepko E., Galotto M.J., Zizovic I. (2022). Supercritical Foaming and Impregnation of Polycaprolactone and Polycaprolactone-Hydroxyapatite Composites with Carvacrol. Processes.

[104] Bendicho-Lavilla C., Seoane-Viaño I., Santos-Rosales V., Díaz-Tomé V., Carracedo-Pérez M., Luzardo-Álvarez A.M., García-González C.A., Otero-Espinar F.J. (2023). Intravitreal Implants Manufactured by Supercritical Foaming for Treating Retinal Diseases. J. Control. Release.

[105] Kravanja K.A., Xhanari K., Knez Marevci M., Maver U., Finšgar M. (2024). Ketoprofen-Loaded PLGA-Based Bioactive Coating Prepared by Supercritical Foaming on a TiAl6V4 Substrate for Local Drug Delivery in Orthopedic Applications. Prog. Org. Coat..

[106] Rivera P., Torres A., Romero J., Alarcón Á., Martínez S., Arrieta M.P., Rodríguez-Mercado F., Galotto M.J. (2024). Effect of Operational Variables on Supercritical Foaming of Caffeic Acid-Loaded Poly(Lactic Acid)/Poly(Butylene Adipate-Co-Terephthalate) Blends for the Development of Sustainable Materials. Polymers.

[107] Freiberg S., Zhu X.X. (2004). Polymer Microspheres for Controlled Drug Release. Int. J. Pharm..

[108] Pulingam T., Foroozandeh P., Chuah J.-A., Sudesh K. (2022). Exploring Various Techniques for the Chemical and Biological Synthesis of Polymeric Nanoparticles. Nanomaterials.

[109] Adhikari C. (2021). Polymer Nanoparticles-Preparations, Applications and Future Insights: A Concise Review. Polym.-Plast. Technol. Mater..

[110] Kumar R., Thakur A.K., Kali G., Pitchaiah K.C., Arya R.K., Kulabhi A. (2023). Particle Preparation of Pharmaceutical Compounds Using Supercritical Antisolvent Process: Current Status and Future Perspectives. Drug Deliv. Transl. Res..

[111] Liu H., Liang X., Peng Y., Liu G., Cheng H. (2024). Supercritical Fluids: An Innovative Strategy for Drug Development. Bioengineering.

[112] Franco P., De Marco I. (2020). Supercritical Antisolvent Process for Pharmaceutical Applications: A Review. Processes.

[113] Prosapio V., De Marco I., Reverchon E. (2018). Supercritical Antisolvent Coprecipitation Mechanisms. J. Supercrit. Fluids.

[114] Ozkan G., Franco P., De Marco I., Capanoglu E., Esatbeyoglu T. (2022). Investigating the Effects of Supercritical Antisolvent Process and Food Models on Antioxidant Capacity, Bioaccessibility and Transepithelial Transport of Quercetin and Rutin. Food Funct..

[115] Wu H.-T., Tu Y.-J., Huang Y.-X., Ng K.H. (2025). Monodisperse Protein Nanoparticles Production by Supercritical Assisted Atomization. J. Supercrit. Fluids.

[116] Wu H.-T., Chuang Y.-H., Lin H.-C., Hu T.-C., Tu Y.-J., Chien L.-J. (2022). Immediate Release Formulation of Inhaled Beclomethasone Dipropionate-Hydroxypropyl-Beta-Cyclodextrin Composite Particles Produced Using Supercritical Assisted Atomization. Polymers.

[117] Wang X., He S., Wang K., Wang X., Yan T., Yan T., Wang Z. (2023). Fabrication of Betamethasone Micro- and Nanoparticles Using Supercritical Antisolvent Technology: In Vitro Drug Release Study and Caco-2 Cell Cytotoxicity Evaluation. Eur. J. Pharm. Sci..

[118] Lu X.-C., Chen B.-Q., Li S.-Q., Xu J.-F., Kankala R.K., Wang S.-B., Chen A.-Z. (2025). Preparation of Hesperetin-Polyvinylpyrrolidone Sub-Microparticles by Supercritical Anti-Solvent Technique for Improved Anti-Cancer Efficiency. J. Supercrit. Fluids.

[119] Cerro D., Cabañas A., Torres A., Zahran F., Sepúlveda L., Rivera P., Romero J. (2026). Precipitation and Encapsulation of β-Sitosterol Using Supercritical Antisolvent (SAS) Method for Controlled Nutraceutical Release. Food Res. Int..

[120] Vallejo R., Quinteros D., Gutiérrez J., Martínez S., Rodríguez Rojo S., Ignacio Tártara L., Palma S., Javier Arias F. (2024). Acetazolamide Encapsulation in Elastin like Recombinamers Using a Supercritical Antisolvent (SAS) Process for Glaucoma Treatment. Int. J. Pharm..

[121] Wang Y., Li Z., Li F., Weiqiang W. (2025). Application Study on the Preparation of Curcumin/PVP Coprecipitated Particles Using Coaxial Nozzle in Supercritical Antisolvent. ACS Omega.

[122] Iannaco M.C., Mottola S., De Marco I. (2026). Engineering Acyclovir-PVP Coprecipitated Microparticles Obtained via Different Supercritical Processes. J. Supercrit. Fluids.

[123] Baldino L., Sarnelli S., Palazzo I., Scognamiglio M., Reverchon E. (2025). Production of Cannabidiol Nanoparticles Loaded in Polyvinylpyrrolidone Microparticles by Supercritical CO_2_ Assisted Atomization and Dissolution Enhancement Effect. Adv. Powder Technol..

[124] Baldino L., Sarnelli S., Scognamiglio M., Reverchon E. (2024). Production of Biopolymeric Microparticles to Improve Cannabigerol Bioavailability. Materials.

[125] Wu H.-T., Tu Y.-J., Jhang R.-S., Ng K.H., Chien L.-J. (2026). Inhalable Hydroxychloroquine−albumin Nanoparticles with Sustained-Release Profiles Prepared via Supercritical Assisted Atomization. J. Supercrit. Fluids.

[126] Wu H.-T., Lin H.-C., Tu Y.-J., Ng K.H. (2023). Instant Formulation of Inhalable Beclomethasone Dipropionate—Gamma-Cyclodextrin Composite Particles Produced Using Supercritical Assisted Atomization. Pharmaceutics.

[127] Lima A.L., Gratieri T., Cunha-Filho M., Gelfuso G.M. (2022). Polymeric Nanocapsules: A Review on Design and Production Methods for Pharmaceutical Purpose. Methods.

[128] Da Silva R.Y.P., De Menezes D.L.B., Oliveira V.D.S., Converti A., De Lima Á.A.N. (2023). Microparticles in the Development and Improvement of Pharmaceutical Formulations: An Analysis of In Vitro and In Vivo Studies. Int. J. Mol. Sci..

[129] Sharma K., Porat Z., Gedanken A. (2021). Designing Natural Polymer-Based Capsules and Spheres for Biomedical Applications—A Review. Polymers.

[130] Yan C., Kim S.-R. (2024). Microencapsulation for Pharmaceutical Applications: A Review. ACS Appl. Bio Mater..

[131] Marin E., Tapeinos C., Sarasua J.R., Larrañaga A. (2022). Exploiting the Layer-by-Layer Nanoarchitectonics for the Fabrication of Polymer Capsules: A Toolbox to Provide Multifunctional Properties to Target Complex Pathologies. Adv. Colloid Interface Sci..

[132] Van Der Kooij R.S., Steendam R., Frijlink H.W., Hinrichs W.L.J. (2022). An Overview of the Production Methods for Core–Shell Microspheres for Parenteral Controlled Drug Delivery. Eur. J. Pharm. Biopharm..

[133] Duran M., Serrano A., Nikulin A., Dauvergne J.-L., Derzsi L., Palomo Del Barrio E. (2022). Microcapsule Production by Droplet Microfluidics: A Review from the Material Science Approach. Mater. Des..

[134] Park H., Kim J.-S., Kim S., Ha E.-S., Kim M.-S., Hwang S.-J. (2021). Pharmaceutical Applications of Supercritical Fluid Extraction of Emulsions for Micro-/Nanoparticle Formation. Pharmaceutics.

[135] Kim J.-W., Han S.H., Choi Y.H., Hamonangan W.M., Oh Y., Kim S.-H. (2022). Recent Advances in the Microfluidic Production of Functional Microcapsules by Multiple-Emulsion Templating. Lab Chip.

[136] Palazzo I., Reverchon E. (2023). Testing the Encapsulation of Phase Change Materials Using Supercritical Emulsion Extraction. J. Supercrit. Fluids.

[137] Kravanja K.A., Finšgar M., Knez Ž., Knez Marevci M. (2022). Supercritical Fluid Technologies for the Incorporation of Synthetic and Natural Active Compounds into Materials for Drug Formulation and Delivery. Pharmaceutics.

[138] Park J.I., Saffari A., Kumar S., Günther A., Kumacheva E. (2010). Microfluidic Synthesis of Polymer and Inorganic Particulate Materials. Annu. Rev. Mater. Res..

[139] El Itawi H., Fadlallah S., Perré P., Allais F. (2023). Microfluidics for Polymer Microparticles: Opinion on Sustainability and Scalability. Sustain. Chem..

[140] Porta G.D., Falco N., Reverchon E. (2011). Continuous Supercritical Emulsions Extraction: A New Technology for Biopolymer Microparticles Production. Biotechnol. Bioeng..

[141] Rezvantalab S., Keshavarz Moraveji M. (2019). Microfluidic Assisted Synthesis of PLGA Drug Delivery Systems. RSC Adv..

[142] Park H. (2024). Exploring the Effects of Process Parameters during W/O/W Emulsion Preparation and Supercritical Fluid Extraction on the Protein Encapsulation and Release Properties of PLGA Microspheres. Pharmaceutics.

[143] Liu F., Su R., Li Y., Ji Y. (2025). Preparation of Hydrophobic Drug Microcapsules via an Intensified Supercritical Extraction of Emulsion Method: Phase Behavior Investigation and Emulsion Formula Regulation. Adv. Powder Technol..

[144] Iannaco M.C., Mottola S., De Marco I. (2026). Emulsification-Driven Size Control in Supercritical PLGA-Based Encapsulation: From Nanocapsules to Microcapsules. Powder Technol..

[145] Vizcaya D., Tirado D.F., Cabañas A., Serrano D.R., Calvo L. (2026). Preparation of 5-ASA-Loaded Eudragit^®^ S100 Nanoparticles by Emulsion-Based Methods: Comparison between Solvent Evaporation and Supercritical Fluid Extraction. J. Supercrit. Fluids.

[146] Park H., Ha E., Kim J., Kim M. (2023). Injectable Sustained-release Poly(Lactic-co-glycolic Acid) (PLGA) Microspheres of Exenatide Prepared by Supercritical Fluid Extraction of Emulsion Process Based on a Design of Experiment Approach. Bioeng. Transla Med..

